# Synthesis, Characterization, and Anticancer Activity of Vanadium (III) Complexes With Pyridyl–Triazole Ligands

**DOI:** 10.1155/bca/2823543

**Published:** 2026-06-15

**Authors:** Yair Alvarez-Ricardo, Deissy N. Jaramillo, Adrian Leonardo Orjuela, Elizabeth Jiménez-Díaz, Jorge Alí-Torres, Mario A. Macías, Antonino Arenaza-Corona, David Morales-Morales, John J. Hurtado

**Affiliations:** ^1^ Research Group in Inorganic Chemistry, Catalysis and Bioinorganic Chemistry, Department of Chemistry, Universidad de Los Andes, 1st Avenue No. 18A-12, Bogota, 111711, Colombia, uandes.cl; ^2^ Applied Biochemistry Research Group (GIBA), Department of Chemistry, Universidad de Los Andes, 1st Avenue No. 18A-12, Bogota, 111711, Colombia, uandes.cl; ^3^ Institute of Scientific Research and High Technology Services (INDICASAT AIP), Panama City, Panama; ^4^ Department of Chemistry, Universidad Nacional de Colombia—Bogota Campus, Bogota, Colombia; ^5^ Crystallography and Chemistry of Materials, CrisQuimMat, Department of Chemistry, Universidad de Los Andes, 1st Avenue No. 18A-12, Bogota, 111711, Colombia, uandes.cl; ^6^ Institute of Chemistry, Universidad Nacional Autónoma de México, Outer Circuit University City, Mexico City, C.P. 04510, Mexico, unam.mx

**Keywords:** anticancer activity, apoptosis, crystal structure, DFT calculations, molecular docking, triazole ligands, V(III) complexes

## Abstract

Three new V (III) complexes with triazole ligands were obtained and fully characterized using regular analytical techniques, including the unequivocal determination of the structure of complex **VL**_**3**_ **D** by single‐crystal x‐ray diffraction analysis. The formation of **VL**_**3**_ **D** involves an oxidation process from V (III) to V (IV) that can occur under conditions similar to those of biological assays. The biological activity of these complexes was explored on six human cancer cell lines exhibiting lower IC50 values in five of them when compared to cisplatin, noteworthy the fact that complex **VL**_**2**_ probed to be more effective against A549 (IC_50_ = 1.07 μM). At the same time, **VL**_**1**_ showed remarkable activity on HCT‐15 (IC_50_ = 3.14 μM). Interestingly and in contrast to cisplatin, the vanadium complexes showed little effect on the healthy fibroblast cells. Mechanistic studies of cell death revealed that **VL**_**3**_ selectively induced late‐stage apoptosis without promoting necrosis in A549 cells, whereas **VL**_**2**_ did not significantly affect apoptotic or necrotic pathways under the conditions tested. These results were complemented with in silico studies and displacement assays, demonstrating the affinity of the complexes for DNA. The results presented in this work are relevant, as there are few reports of similar V (III) compounds explored as potential anticancer agents.

## 1. Introduction

Cancer remains one of the most pressing global health concerns, accounting for a significant number of deaths each year. In 2022, according to data from the World Health Organization (WHO), approximately one in six individuals diagnosed with this disease succumbed to its effects, totaling approximately 10 million deaths worldwide [1].

In response to this challenge, the synthesis of metal complexes has emerged as a promising approach, with platinum‐based compounds being particularly notable. Among these, cisplatin stands out as one of the most prominent and widely utilized chemotherapeutic agents. This compound has played a crucial role in the treatment of various malignancies, including lung, ovarian, bladder, and testicular cancers. Its mechanism of action involves the inhibition of DNA replication in cancer cells, rendering it as a potent therapeutic agent. However, a major limitation of platinum‐based compounds, as well as other chemotherapeutic agents, is their lack of selectivity. This indiscriminate action results in cytotoxic effects not only on malignant cells but also on healthy tissues, leading to significant adverse effects. Common side effects include anemia, fatigue, renal and urinary complications, alopecia, appetite changes, nausea, and vomiting, among others [2]. Notably, immunosuppression is of particular concern, as it increases susceptibility to infections and further complicates treatment. These challenges have prompted the search for alternative compounds with improved selectivity and efficacy. Among these, vanadium‐based complexes have garnered significant interest, as they have demonstrated comparable or even superior cytotoxic effects against cancer cells. These compounds have shown promising activity across various cancer models, including neuroblastoma (SK‐N‐SH and SH‐SY5Y), breast cancer, colon cancer, and pancreatic cancer, among others [3].

Vanadium has attracted considerable attention in biomedical research due to its well‐documented role as an insulin‐mimetic agent. The mechanisms explored in the context of diabetes treatment have opened new avenues for cancer therapy, particularly through the modulation of caspase signaling pathways involved in apoptosis, or programmed cell death [4]. In a study conducted by Narla et al., a vanadium complex was evaluated in leukemia, demonstrating its capacity to prevent the aggregation of malignant cells in the bone marrow. Furthermore, this compound exhibited significantly greater efficacy in inducing apoptosis in primary leukemic cells compared to standard chemotherapeutic agents such as dexamethasone and vincristine [5].

In parallel, N‐heterocyclic compounds have been extensively studied due to their diverse pharmacological properties, including anticancer, anti‐HIV, antimicrobial, and antidiabetic activities, and they have been molecules of our research interest. The presence of nitrogen atoms within these structures facilitates hydrogen bonding with biological targets, enhancing their bioactivity [6–9]. Among this class of compounds, azoles are particularly noteworthy, serving as key scaffolds for numerous biologically active derivatives, particularly antifungal agents. Structural and functional similarities between fungal components and certain cancer cell characteristics have led to increasing interest in the potential repurposing of antifungal agents for cancer treatment, particularly in advanced disease stages where conventional therapies exhibit limited efficacy [10].

Wang et al. investigated the anticancer potential of itraconazole, a triazole derivative with well‐established antifungal properties, in MCF‐7 and SKBR‐3 breast cancer cells. Following a 12‐h incubation period, they observed cell cycle arrest and cell death, primarily mediated through apoptosis, as evidenced by increased caspase activity. Additionally, itraconazole induced autophagic cell death, a process that maintains membrane integrity until the final stages of cell death, thereby preventing the release of intracellular components and minimizing inflammatory responses [11]. The efficacy of itraconazole was further evaluated in a murine model, where it demonstrated significant anticancer activity against breast cancer. Specifically, a reduction in tumor volume was observed, alongside increased apoptosis and autophagy levels within the tumor tissue [11]. Similarly, studies conducted by Yuan et al. assessed the potential of miconazole, an azole derivative commonly used for the treatment of vaginal fungal infections, in bladder cancer. Their findings revealed that miconazole effectively inhibited the proliferation of cancer cells (T24 and TSGH‐8301) by inducing apoptosis. Furthermore, miconazole treatment resulted in internucleosomal DNA fragmentation, a hallmark of apoptotic cell death [12].

Beyond these investigations, numerous studies have documented the application of azole‐based compounds, renowned for their antifungal efficacy, in the treatment of various malignancies. Furthermore, the therapeutic potential of metal‐based complexes in oncology has been increasingly recognized. Considering these findings, the present study aimed to synthesize three azole‐derived ligands and their corresponding V (III) complexes to determine their potential anticancer activity.

## 2. Materials and Methods

### 2.1. General Information

All chemicals were available commercially, and the reagent‐grade solvents were dried and distilled before use. Melting points were determined on a Mel‐Temp 1101D apparatus and are reported uncorrected. Mass spectra were obtained using a JEOL SX 102A spectrometer on Bruker Microflex equipped with MALDI–Flight time. Elemental analysis (C, H, and N) was performed with a Thermo Scientific FLASH 2000 CHNS/O Analyzer. Fourier transform infrared (FT‐IR) spectra were recorded on a Thermo Nicolet NEXUS FT‐IR spectrophotometer using the ATR module. Raman spectroscopy was performed in a RIBA Yovin‐Ion spectrometer using two lasers with 638 nm wavelengths. Ultraviolet/visible (UV/vis) spectra were recorded on an Agilent Technologies Cary 100 spectrophotometer in DMSO from 200 to 900 nm in a quartz cuvette with a path length of 1 cm. Thermogravimetric analysis (TGA) analyses of the complexes were conducted on a NETZSCH STA 409 PC/PG by evaluating 7–10‐mg samples of the complexes in a nitrogen atmosphere. Samples were subjected to dynamic heating over a temperature range of 30°C–700°C at a heating rate of 10°C/min. The ligands 2,6‐bis((1H‐1,2,4‐triazol‐1‐yl)methyl)pyridine (**L**_**1**_), 2,6‐bis((1H‐benzo[d][1,2,3]triazol‐1‐yl)methyl)pyridine (**L**_**2**_), and pyridine‐2,6‐diylbis((1H‐benzo[d][1,2,3]triazol‐1‐yl)methanone) (**L**_**3**_) were prepared according to literature methods [7, 13] and confirmed by ^1^H NMR (Figure S1–S3) in a Bruker AscendTM‐400 spectrometer at 295K, and chemical shifts are reported in ppm relative to DMSO‐*d*_6_.

### 2.2. Synthesis of Vanadium Complexes

#### 2.2.1. Synthesis of 2,6‐bis((1H‐1,2,4‐triazol‐1‐yl)methyl)pyridine‐VCl_3_ (**VL**_**1**_)

0.066 g of VCl_3_ was dissolved in 10 mL of tetrahydrofuran (THF) in a 100‐mL Schlenk flask at 65°C, to which a solution of 0.100 g of **L**_**1**_, previously dissolved in 10 mL of THF, was slowly under stirring. The resulting reaction mixture was kept under stirring for 24 h. After this time, the solid formed was filtered, washed with THF and ethyl ether, and finally dried under vacuum. Yield: 67%; m.p. 230°C. MALDI mass spectrum: [M^+^]: *m/z* 396.19, [M + H^+^]: 397.19. Anal. Calc. for C_11_H_11_Cl_3_N_7_V: C, 33.15; H, 2.78; N, 24.60. Found: C, 33.21; H, 2.78; N, 24.63. IR (ATR cm^−1^): (C=N) 1527, (C–N triazole) 1122, (N‐N) 1276 cm^−1^. Raman (cm^−1^): 93.32, 134.90, 186.67, 281.58.

#### 2.2.2. Synthesis of 2,6‐bis((1H‐benzo[d][1,2,3]triazol‐1‐yl)methyl)pyridine‐VCl_3_ (**VL**_**2**_)

**VL**_**2**_ was prepared in the same manner as **VL**_**1**_, using 0.252 g of **L2** with 0.127 mg of VCl_3_ employing THF as dissolvent. Yield: 50%; m.p. 255°C. MALDI mass spectrum: [M^+^]: *m/z* 496.39, [M + H^+^]: 397.39. Anal. Calc. for C_19_H_15_Cl_3_N_7_V: C, 45.76; H, 3.03; N, 19.66. Found: C, 45.83; H, 3.05; N, 19.39. IR (ATR cm^−1^): (C=N) 1531, (N‐N) 1083. Raman (cm^−1^): 86.55, 130.80, 178.46, 311.08.

#### 2.2.3. Synthesis of Pyridine‐2,6‐diylbis((1H‐benzo[d][1,2,3]triazol‐1‐yl)methanone)‐VCl_3_ (**VL**_**3**_)

Using the same procedure as above, 0.311 g of **L**_**3**_ was added to a mixture of 0.146 g of VCl_3_ dissolved in THF. Yield: 54%; m.p. 160°C. MALDI mass spectrum: [M + Na–Cl]^+^: *m/z* 512.09. Anal. Calc. for C_19_H_15_Cl_3_N_7_V: C, 43.33; H, 2.11; N, 18.62. Found: C, 43.35; H, 2.15; N, 18.67. IR (ATR cm^−1^): (C=O, amide) 1674, (C=N) 1604, (N‐N) 1049. Raman (cm^−1^): 101.93, 137.91, 196.09, 277.73. **VL**_**3**_ was placed in an ethanol–water mixture at room temperature, and by slow evaporation, crystals suitable for XRD characterization were obtained, which led to its derivative, named **VL**_**3**_ **D**.

### 2.3. DFT Calculations

To evaluate the anticancer potential of the vanadium complexes in the DNA pathway, we investigated the molecular complexes **VL**_**1**_, **VL**_**2**_, and **VL**_**3**_. We explored possible conformers using AutoDE [14] and CREST [15], followed by energy evaluation with the xTB method [16]. Before conformer exploration, geometry optimization was conducted, and stationary points were characterized through frequency calculations. These DFT studies were performed using Gaussian 16 [17], employing the B3LYP functional with the 6–31 + G (d, p) basis set for all atoms. Finally, we calculated NBO charges to use in molecular docking studies [18].

For the molecular docking studies, we used a DNA structure (PDB code: 1AIO) [19] as the target, chosen because it is commonly used in studies with cisplatin, a well‐known cancer therapy agent. The target was prepared with AutoDock Tools 4 [20], and Gasteiger charges were assigned to this receptor [21]. The most stable vanadium complexes were selected for the molecular docking protocol. Due to the lack of specific parameters for vanadium in common molecular docking programs, we used the AutoDock4 package to explore the binding site of these vanadium complexes [20]. The results were analyzed using Maestro [22] and Chimera software [23].

To study the electronic structure of the crystal in a MeOH/water solvent, we applied the same electronic structure methods, incorporating Grimme dispersion and Becke–Johnson corrections [24]. We then calculated the free energy, considering solvent effects using the SMD [25], to corroborate the exergonicity of the process.

To explore the impact of adding water molecules, we employed the xTB semiempirical method to analyze structural changes in the **VL**_**3**_ derivative complex [16]. Before this, we utilized electronic structure methods to confirm the geometry and electronic environment around the metallic center. Additionally, we applied the APOST‐3D approach to examine changes in the oxidation state of vanadium [26, 27].

### 2.4. Crystallographic Analysis

The x‐ray intensity data of the **VL**_**3**_ **D** complex were measured at room temperature, 298 (2) K, using CuKα (*λ* = 1.54184 Å), through *ω* scans, in an Agilent SuperNova, Dual, Cu at Zero, Atlas four‐circle diffractometer equipped with a CCD plate detector. The collected frames were integrated and corrected for the absorption effect using the CrysAlis PRO software package (CrysAlisPro 1.171.39.46e, Rigaku Oxford Diffraction, 2018). The crystal structure was solved using an iterative algorithm [28] and completed by a difference Fourier map. All the nonhydrogen atoms were refined anisotropically, while the hydrogen atoms were generated geometrically and placed in calculated positions. The crystal structure was refined using the program SHELXL2018/3 [29]. Molecular and supramolecular graphics were carried out using the software Mercury [30]. Crystallographic data have been deposited in the Cambridge Crystallographic Data Center (CCDC) with a Deposition No. CCDC‐2442598. Hirshfeld (HF) surface analysis mapped over d_norm_ was performed using the models implemented in CrystalExplorer [31, 32].

### 2.5. Cytotoxic Assays

The cytotoxicity of all synthesized compounds was evaluated against a panel of seven human cancer cell lines: U251 (glioblastoma), PC3 (prostate adenocarcinoma), K562 (chronic myelogenous leukemia), HCT‐15 (colon adenocarcinoma), MCF‐7 (mammary adenocarcinoma), SK‐LU‐1 (lung adenocarcinoma), and A549 (lung carcinoma). Cytotoxicity analyses were also performed in the L929 murine fibroblast cell line to assess compound safety and selectivity, representing noncancerous cells. All cell lines were obtained from the U.S. National Cancer Institute (NCI).

Cells were cultured in RPMI‐1640 medium or DMEM supplemented with 10% fetal bovine serum (FBS), 2 mM L glutamine, 10,000 U/mL penicillin *G* sodium, 10,000 μg/mL streptomycin sulfate, 25 μg/mL amphotericin B (Invitrogen/Gibco, Thermo Fisher Scientific, Waltham, MA, USA), and 1% nonessential amino acids (Gibco). Cultures were maintained at 37°C in a humidified atmosphere containing 5% CO_2_. Only cell populations with viability above 95%, as determined by trypan blue exclusion, were used in the experiments.

Cytotoxicity was assessed after 72 h of compound exposure using the sulforhodamine B (SRB) colorimetric assay, following the standardized protocols established by the NCI [33–35]. All test compounds were dissolved in DMSO, with the final DMSO concentration in assay wells kept below 0.1%, a level shown experimentally to have no impact on cell viability. Medium‐only (RPMI or DMEM) and DMSO (25% v/v) controls were used as negative and positive controls for cytotoxicity, respectively.

The results were reported as mean inhibitory concentration values (IC_50_), defined as the concentration required to inhibit cell growth by 50%. IC_50_ values were determined by fitting the dose–response curves using nonlinear regression analysis, as described by Monks et al. [33]. The selectivity index (SI) was calculated as the ratio of the IC_50_ value in normal cells (L929) to that in cancer cells, providing a measure of therapeutic selectivity. All assays were performed in quintuplicate across three independent experiments to ensure reproducibility.

### 2.6. Ethidium Bromide Displacement Assay

Ethidium bromide displacement assays were performed using a Varioskan LUX Multimode Microplate Reader with a 384‐well plate. An excitation wavelength of 450 nm was used, and emission was recorded in the range of 500–840 nm. The excitation bandwidth and step size were set to 12 nm and 10 nm, respectively. A solution containing 3.5 μM of purified double‐stranded DNA (dsDNA 16S–23S ITS region) and 25 μM of ethidium bromide (EtBr) was prepared. First, the fluorescence spectra of EtBr and the DNA/EtBr complex were recorded in the absence of the vanadium complex. Then, 0.5 μL portions of the **VL**_**2**_ complex (10 mM in 50% DMSO) were added stepwise, and after thorough shaking, the fluorescence intensity was measured. Spectra were recorded 1 minute after each addition.

The fluorescence area under the curve was calculated and converted to the percentage of the EtBr displacement value using the equation: *E* *t* *B* *r* *d* *i* *s* *p* *l* *a* *c* *e* *m* *e* *n* *t* (%) = 100 − [(*f*/*f*_0_) × 100, where *f* is the fluorescence area without a ligand and *f_0_* is the fluorescence area in the presence of a complex [36]. The mean displacement concentration value (DC50), defined as the concentration of the added compound required to reduce the fluorescence of the EtBr/DNA complex to 50% [37], was determined by fitting dose–response curve using nonlinear regression analysis in *R* software (v4.4.2; *R* Core Team, 2024) with the *drc* package. All spectra were analyzed using *Origin* 9.0 software.

### 2.7. Flow Cytometry Analysis

To determine the mechanism of cell death induced by **VL**_**2**_ and **VL**_**3**_, phosphatidylserine exposure was assessed using Annexin V–FITC/PI Apoptosis Detection Kit (BioLegend Way, San Diego, CA), according to the manufacturer’s instructions. Flow cytometric analysis was performed using a BD Accuri C6 flow cytometer (BD Biosciences, San Jose, CA, USA), with 30,000 events acquired per sample. Annexin V exhibits high affinity for phosphatidylserine, enabling the identification of cells at different stages of apoptosis. Propidium iodide (PI) was employed to identify late apoptotic and dead cells by selectively staining cells with compromised membrane integrity. Untreated cells were used as the negative control, while doxorubicin served as the positive control to validate the assay. Data analysis was performed using Floreada free software (Floreada.io).

### 2.8. Statistical Analysis

All data are presented as the arithmetic mean ± standard error of the mean (SEM). Statistical analyses were performed using Origin 2022b software (OriginLab Corporation, Northampton, MA, USA). Differences in cytotoxic activity in normal cells were assessed using the nonparametric Kruskal–Wallis test followed by Dunn’s post hoc test. Cell death data were analyzed using the two‐way analysis of variance (ANOVA) followed by Tukey’s post hoc test. A *p* value of less than 0.05 was considered statistically significant.

### 2.9. Methodology to Calculate LogP

The lipophilicity of the ligands and vanadium complexes was estimated by calculating the octanol–water partition coefficient (logP) using the RDKit cheminformatics toolkit (Version 2023.09). The molecular structures were defined using SMILES notation, and the logP values were computed employing the Crippen atom‐based fragment method as implemented in RDKit through the MolLogP descriptor [38, 39].

## 3. Results and Discussion

### 3.1. Synthesis and Characterization of VL_1_–VL_3_

**SCHEME 1:**
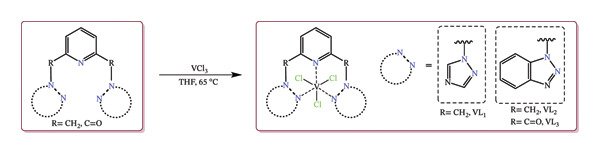
Synthesis of vanadium (III) complexes of **L**_**1**_ **–L**_**3**_.

Ligands **L**_**1**_ **–L**_**3**_ were prepared according to previously published procedures by our research group [13] and confirmed by ^1^H NMR (Figure S1 – S3). And complexes were produced by the addition of the ligands to a solution of VCl_3_ in THF under stirring at 65°C (Scheme 1).

Previously, two complexes of 3,5‐dimethylpyrazole with VCl_3_ were reported by Abbo et al., [40] establishing the formation of V (III) chloride complexes having the N, N, N ligand coordinated in a tridentate manner through IR spectroscopy and mass spectrometry techniques, where in this last technique, the fragmentation pattern for their complexes was the loss of VCl_3_; this same profile was observed in our compounds (Figures S14 – S16). These findings were later confirmed by Zhang et al., [41] using similar reaction conditions to synthesize three analogous complexes, one of which was structurally characterized by crystallographic techniques.

Building on these previous findings, FT‐IR analysis of our complexes revealed shifts in the bands when compared to the free ligands. For the C–N stretching in the triazoles, the band in the free ligand **L**_**1**_ was observed at 1141 cm^−1^, whereas in **VL**_**1**_, it was found at 1122 cm^−1^. Similarly, the N–N stretching band appeared at 1269 cm^−1^ in **L**_**1**_ and at 1276 cm^−1^ in **VL**_**1**_. These shifts suggest coordination between vanadium and nitrogen at Position 2 of both 1,2,4‐triazoles in the ligands.

Additionally, the third coordination site with the ligand involves the nitrogen of the pyridine. Based on the FT‐IR spectrum analysis, it can be proposed that this is a tridentate complex, as the C=N stretching band in **L**_**1**_ was identified at 1508 cm^−1^, while in **VL**_**1**_, it was observed at 1527 cm^−1^.

In the case of **L**_**2**_ and **L**_**3**_, the bands corresponding to the C=N stretching were observed at 1492 and 1485 cm^−1^, respectively. In contrast, in **VL**_**2**_ and **VL**_**3**_, these bands appeared at 1531 and 1604 cm^−1^, showing an increase in the intensity compared to the free ligand. The N–N stretching vibrations of the pyrazole ring were observed at 1087 cm^−1^ in **L**_**2**_ and at 1049 cm^−1^ in **L**_**3**_. However, in **VL**_**2**_ and **VL**_**3**_, this band was found at 1083 and 1076 cm^−1^, respectively, with a notable decrease in the intensity. These changes in spectral shifts and intensities in FT‐IR suggest that vanadium is coordinated to both the pyridine’s and the pyrazole ring’s nitrogen (Figures S4–S9).

Raman spectra of the starting salt VCl_3_ showed four bands between 96.46 and 281.47 cm^−1^ associated with V–Cl [42], while for **VL**_**1**_ **–VL**_**3**_, the corresponding bands were observed between 93.32 and 281.58, 86.55–311.08, and 101.93–277.73 cm^−1^, respectively, with notorious changes in displacements and intensities of the bands (Figures S10–S13).

Besides, UV–vis spectra of the free ligands in DMSO displayed a band at 269 nm for **L**_**1**_, two bands at 266 and 284 nm for **L**_**2**_ **,** and two bands at 268 and 305 nm for **L**_**3**_ (Figures S17–S19). And in the case of the complexes, these bands were found at 270 nm for **VL**_**1**_, at 265 and 283 nm for **VL**_**2**_, while bands at 262 and 283 nm were identified for **VL**_**3**_ (Figure 7). This suggests that the complexes containing benzotriazoles in their structure experienced a hypsochromic shift, whereas the complex with triazole showed a slight bathochromic shift. On the other hand, VCl_3_ exhibited three bands at 271, 463, and 705 nm, corresponding to the transitions ^3^A_2_g (F) ⟵ ^3^T_1_g (F), ^3^T_1_g (P) ⟵ ^3^T_1_g (F), and ^3^T_2_g (F) ⟵ ^3^T_1_g (F), respectively [42]. Given the proximity of the position of the 271‐nm band to those observed in the ligands, it is possible to assume an overlap of these in all metal complexes.

The 705‐nm band found in the metal salt is shifted by 689, 815, and 822 nm for **VL**_**1**_ **–VL**_**3**_, respectively. These bands together with the observed in Raman infer the presence of V (III) in the complexes.

In the case of **VL**_**1**_, the shift is hypsochromic, while **VL**_**2**_ and **VL**_**3**_ are bathochromic. Finally, the 463‐nm band detected in VCl_3_ was only observed in **VL**_**1**_ at 486 nm, indicating a bathochromic shift (Figures S20–S26). Also, TGAs showed that the **VL**_**2**_ complex is the most thermally stable species, followed by **VL**_**1**_ and finally **VL**_**3**_, which correlates with the uncorrected melting points found (Figures S29–S31). Due to the low solubility of the complexes in common solvents, all attempts to obtain single crystals appropriate for x‐ray diffraction were unsuccessful, except a derivative of **VL**_**3**_ named **VL**_**3**_ **D** whose crystallographic results will be discussed in section 3.3. However, the spectroscopy and analytical data are consistent with the proposed formulation for complexes.

On the other hand, based on the background and characterizations, studies were carried out to establish the most stable conformation, i.e., fac or merc, in these octahedral compounds, for which computational studies were performed.

### 3.2. Conformational and Energetic Analysis of Vanadium Complexes

As shown in Table 1, three conformers were obtained for both **VL**_**1**_ and **VL**_**2**_. The first two conformations are **mer** isomers. However, in the lowest‐energy complex, the hydrogen positions on the linker are asymmetric, and the pyridine ring is rotated. For the second complex, the hydrogen positions are symmetric, and the pyridine ring is positioned in the plane. This observation highlights the conformational diversity provided by the flexibility of the methyl group. For the last complex in the **fac** form, the hydrogens exhibit a symmetric arrangement. Based on energy values, for the **VL**_**1**_ and **VL**_**2**_ complexes, the most stable conformation is **mer**.

**TABLE 1:** Conformer optimization at B3LYP/6‐31++G (d,p) with atom color coding: vanadium (light gray), nitrogen (blue), chlorine (green), and hydrogen (white).

VL_1_	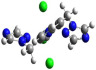	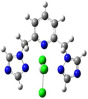	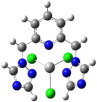
*ΔE* kcal/mol	0	5.39	6.65
VL_2_	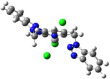	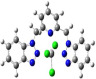	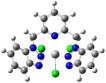
*ΔE* kcal/mol	0	6.34	5.24

**FIGURE 1:**
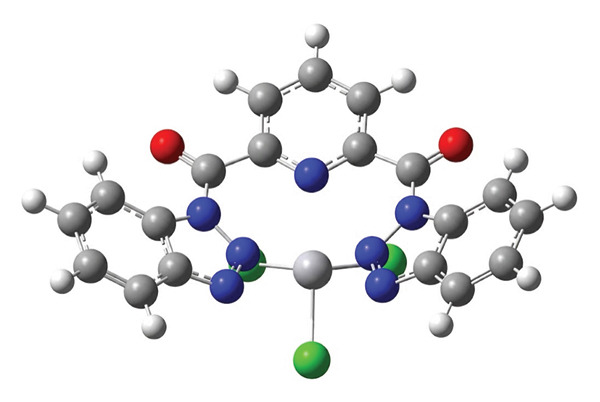
Conformer optimization of **VL**_**3**_ at B3LYP/6‐31++G (d,p) with atom color coding: vanadium (light gray), nitrogen (blue), chlorine (green), oxygen (red), and hydrogen (white).

For the **VL**_**3**_ complex, only one minimum‐energy conformation was obtained. The carbonyl linker (C=O) does not provide the same flexibility as the methyl group, making the ***fac*** conformation the most stable, as shown in Figure 1.

### 3.3. Crystallographic Analysis

**FIGURE 2:**
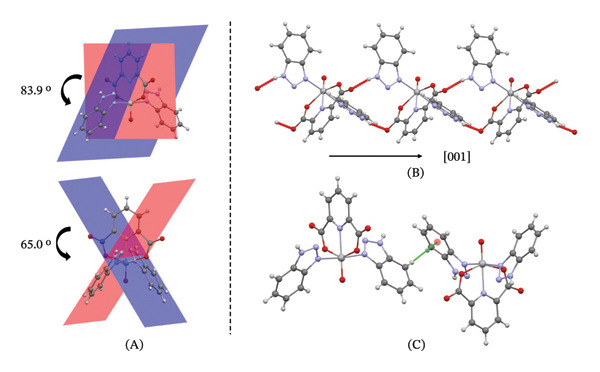
(A) Dihedral angles between pyridine‐dicarboxylate and benzotriazole fragments (up) and benzotriazole–benzotriazole fragments (down). (B) N–H‧‧‧O hydrogen bonds (symmetry code: x, y, 1 + *z*). (C) C–H‧‧‧*π* hydrogen interactions (symmetry code: 3/2 − x, 1 − y, 1/2 + *z*).

The crystal structure of a derivative of complex **VL**_**3**_ was unequivocally determined using single‐crystal x‐ray diffraction techniques (named **VL**_**3**_ **D**). The formation of **VL**_**3**_ **D** involves an oxidation process from V (III) to V (IV) that can occur under conditions like those of biological assays. In the molecular structure, the pyridine‐dicarboxylate fragment is orthogonally oriented to the benzotriazole fragments by a dihedral angle of 83.9°, being the dihedral angle between both benzotriazole fragments of 65.0° (Figure 2A). The V^IV^=O bond in the molecule has a bond length of 1.601(4) Å, which was analyzed using Mogul 2024.1.0 software (CCDC) to validate the structure through the assessment of the calculated molecule with the complete CSD (Cambridge Structural Database). From the results, it was found that the calculated V^IV^=O bond matches with 49 reported structures with a mean bond length of 1.596 Å and standard deviation 0.021 Å. Considering these results, the V^IV^=O bond is validated. From a supramolecular perspective, the planar benzotriazole rings have an important role in molecular packing. **VL**_**3**_ **D** crystallized in the orthorhombic *Pbna* space group and unit cell with high volume (2022.3 Å^3^). Table S1 presents the crystallographic data from the experiment.

In the crystal structure, short N–H‧‧‧O hydrogen bonds (symmetry code: *x*, y, 1 + *z*) connect molecules along the [001] direction, through H‧‧‧O and N‧‧‧O distances of 1.83 Å and 2.660(4) Å, respectively (Figure 2B). These short distances are related to a high acidic character of the hydrogen atoms in the benzotriazole fragments. Neighboring chains interact through C–H‧‧‧*π* hydrogen interactions (symmetry code: 3/2 − *x*, 1 − *y*, 1/2 + *z*) with H‧‧‧*π* and C‧‧‧*π* distances of 2.89 Å and 3.793(5) Å, respectively (Figure 2C). The coordination sphere of V(IV) is formed by [V^IV^O(dipic) (bztz)] with dipic and bztz being the pyridine‐dicarboxylate and benzotriazole ligands forming an octahedral environment with a polyhedron volume of 10.35 Å^3^, a quadratic elongation of 1.041, and an angle variance of 101.31°, indicating a polyhedral distortion [43, 44].

### 3.4. Evaluation of the Crystal Structure by DFT Calculations

**FIGURE 3:**
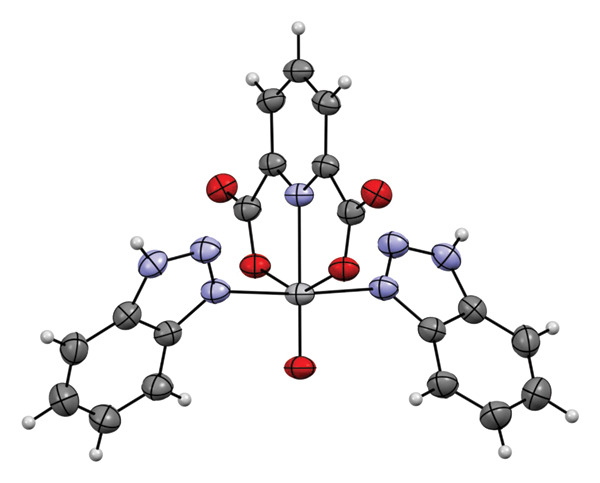
ORTEP with displacement ellipsoids at 50% of probability level from a derivative of **VL**_**3**_ **D.** The V‐ligand bond lengths determined by x‐ray crystallography are V–O: 2.040(2) Å; V=O: 1.601(4) Å; V–N: 2.156(4) Å.

A single crystal of **VL**_**3**_ **D** was obtained when **VL**_**3**_ attempted to be crystallized from methanol/water at room temperature (Figure 3). However, the single crystal suggests that water carried out a C–N bond activation reaction of amides, reactions that have been studied when metals such as Ni, Co, Pd, and Mn are used [45].

**FIGURE 4:**
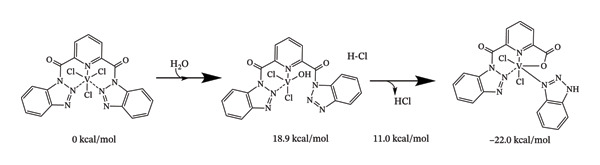
Proposed cyclization process of **VL**_**3**_ in the presence of water, illustrating the formation of the oxo‐vanadium (IV) species (**VL**_**3**_ **D**).

To evaluate the formation of the crystal structure, we initially analyzed the interaction of a water molecule near the vanadium center. Here, the water molecule dissociates into a hydroxyl group and a proton (Figure 4). The hydroxyl group then coordinates with the vanadium center at 1.77 Å, simultaneously causing the V–Cl bond to break through a heterolytic mechanism (Figure S33A). This process results in the dissociation of the triazole ligand, shifting its distance to 4.94 Å. The energy associated with the formation of this intermediate adduct is 18.9 kcal/mol. Meanwhile, the proton reacts to form HCl, which subsequently leaves the coordination sphere, releasing 11.0 kcal/mol of energy.

**FIGURE 5:**
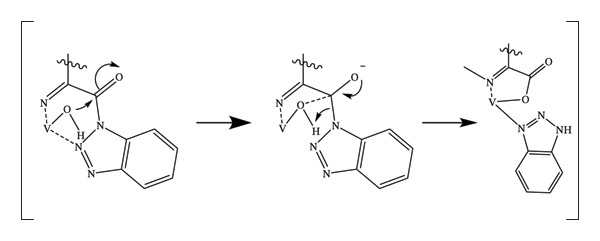
Proposed intramolecular cyclization mechanism leading to the formation of the carboxylic acetal derivative.

The subsequent step involved the formation of a cyclic intermediate resulting from the nucleophilic attack of the hydroxyl group on the carboxyl group (Figure S33B). The proton generated during this attack protonated the triazole ligand, which then coordinated to the vanadium center at 1.90 Å, releasing an energy of −22.0 kcal/mol (Figure 5). The following step repeated this process with an additional water molecule, resulting in the formation and subsequent release of an HCl molecule and yielding an associated energy of −35.8 kcal/mol.

**FIGURE 6:**
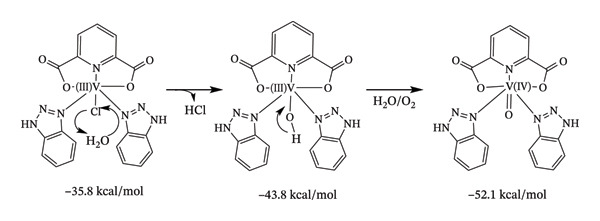
Proposed mechanism for the transformation of **VL**_**3**_ into **VL**_**3**_ **D** under aqueous aerobic conditions. The process involves initial ligand exchange in which the coordinated chloride is replaced by water, generating a V–OH intermediate and releasing HCl. Subsequent oxidation of V(III) to V(IV) leads to the formation of the oxovanadium species **VL**_**3**_ **D** characterized by a V=O bond.

The transformation of **VL**_**3**_ into the oxovanadium species **VL**_**3**_ **D** proceeds through a multistep process involving ligand substitution, proton transfer, and subsequent oxidation under aqueous aerobic conditions (Figure 6). The first step corresponds to a ligand‐exchange process in which the coordinated chloride ligand (Cl^−^) is replaced by a water molecule, leading to the formation of HCl and generating a V(III)–OH intermediate. This substitution step is thermodynamically favorable, with a calculated free energy change of ΔG = −35.8 kcal·mol^−1^.

In the following stage, structural rearrangement and proton transfer within the coordination sphere stabilize the hydroxo intermediate, resulting in an additional decrease in free energy (ΔG = −43.8 kcal·mol^−1^). Finally, the oxidation of the vanadium center occurs in the presence of dissolved molecular oxygen, producing the oxovanadium species characterized by a V(IV)=O bond (Figure S34) with a calculated bond distance of 1.61 Å. This final step is strongly exergonic (ΔG = −52.1 kcal·mol^−1^), confirming that the overall transformation is thermodynamically favorable.

The significant electronic reorganization associated with the oxidation of V (III) to V (IV) and the accompanying change in the spin state was further supported by analysis using the APOST‐3D software package.

### 3.5. Overtime Stability of Metal Complexes by UV–Vis

**FIGURE 7:**
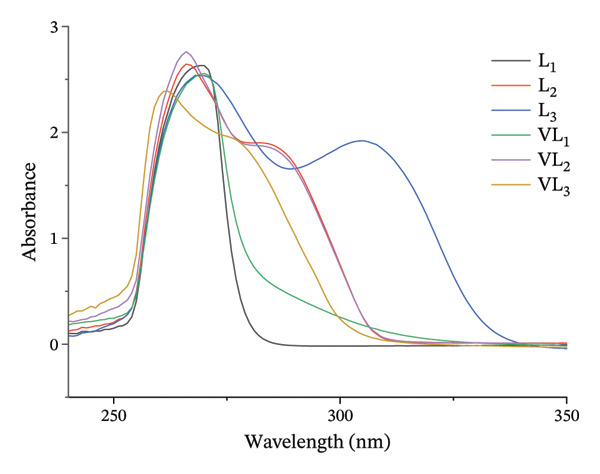
UV–vis spectrum of ligands and their respective metal complexes in DMSO.

As shown in Figure 7, the electronic spectra of the ligands and their corresponding metal complexes were recorded in DMSO. Ligand **L**_**1**_ exhibits an absorption band at 269 nm, which undergoes a slight bathochromic shift to 270 nm upon complexation in **VL**_**1**_, suggesting a minor stabilization of the excited state. In contrast, **L**_**2**_ displays two bands at 266 and 284 nm that experience small hypsochromic shifts to 265 and 283 nm, respectively, in **VL**_**2**_, indicating subtle changes in the electronic distribution upon coordination. Similarly, **L**_**3**_ shows the absorption bands at 268 and 305 nm; however, in **VL**_**3**_, these bands are significantly shifted to 262 and 276 nm, respectively, reflecting more pronounced hypsochromic shifts that may be associated with decreased conjugation or ligand field effects induced by metal coordination.

As can be seen in Figure S27, there are no changes suggesting degradation or modification of the structural characteristics of the complexes in this medium after 4 days. To evaluate the stability of the complexes in an aqueous medium, aliquots corresponding to 5% of the **VL**_**1**_ **–VL**_**3**_ solutions (2.5 × 10^−4^ M) were diluted with biological assay‐grade water to obtain a final volume of 4000 μL. In this mixture, the aqueous phase constituted the remaining 95% (Figure S28).

The stability assays conducted in DMSO and in the H_2_O:DMSO mixture are in good agreement with the cytotoxicity results, as both sets of experiments exhibited consistent and reproducible behavior across different time points, as discussed below.

### 3.6. Cytotoxic Analysis

We tested our vanadium complexes on a carefully selected panel of seven well‐characterized cancer cell lines: MCF‐7 (breast), A549 (lung), PC‐3 (prostate), U251 (glioblastoma), K562 (chronic myeloid leukemia), HCT‐15 (colon), and SK‐LU‐1 (lung adenocarcinoma) to evaluate the antitumor potential across diverse and clinically significant tumor types. These lines were chosen not only for their representation of major histological categories but also for their proven stability and reproducibility in vitro, which supports consistent and comparable results across studies. Additionally, these cell lines have been established for screening agents targeting redox or DNA‐binding mechanisms. For example, MCF‐7 is a prototypical estrogen receptor‐positive breast cancer model harboring hotspot mutation in the PI3K pathway. This strategic selection enabled us to assess both broad‐spectrum cytotoxicity and mechanistic effects in genetically and phenotypically distinctive cancer models, while maintaining high translational relevance. L929 cells were used as a control, a mouse fibroblast cell line recommended by international standards for testing the cytotoxic properties of materials and substances [46]. This cell line is used to evaluate the potential harm of materials and substances by measuring cell viability and morphology after exposure to the substance.

**FIGURE 8:**
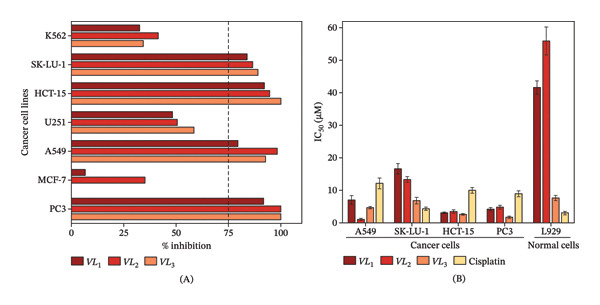
(A) Cytotoxic screening of V (III) complex **VL**_**1**_ **–VL**_**3**_ (25 μM) against six cancer cell lines. (B) IC_50_ values (μM) of vanadium complex **VL**_**1**_ **–VL**_**3**_, and the anticancer drug cisplatin against a panel of human cancer cells and normal cell lines.

According to the above, cytotoxicity assays were performed to evaluate the antiproliferative effects of the V (III) complexes **VL**_**1**_ **–VL**_**3**_ and compared with cisplatin. A single‐dose screening (25 μM) revealed that all complexes significantly inhibited cell growth (> 75%) in PC3, HCT‐15, SK‐LU‐1, and A549 cancer cell lines, suggesting strong antiproliferative potential (Figure 8A). Moderate activity was observed in U251 and K562 cells, while MCF‐7 showed a minimal response (for **VL**_**3**_, the effect is not observed). In contrast, the free ligands **L**_**1**_ **–L**_**3**_ showed no significant effects. Only **L**_**1**_ exhibited inhibition close to 20% in two of the six cancer cell lines (Table S2), highlighting the importance of complexation with vanadium for biological activity. Based on these results, the most responsive cell lines toward vanadium complexes were selected to further determine their IC_50_ values (Figure 8B). **VL**_**3**_ showed potent cytotoxicity across all selected lines, particularly in PC3 (IC_50_ = 1.71 μM) and HCT‐15 (IC_50_ = 2.62 μM). **VL**_**2**_ was the most effective against A549 (IC_50_ = 1.07 μM), while **VL**_**1**_ also displayed notable activity on HCT‐15 (IC_50_ = 3.14 μM). To summarize, all vanadium complexes demonstrated lower IC_50_ values than cisplatin in five of the six tested cancer lines, with SK‐LU‐1 being the least responsive (Table 2, Figure S32).

**TABLE 2:** IC_50_ values (μM) of the V (III) and the anticancer drug cisplatin against a panel of human cancer cells for 72 h, namely, lung (A549 and SK‐LU‐1), colorectal (HCT‐15), and prostate carcinoma (PC3) cells. Murine fibroblasts (L929) were used as control cells.

Compound	Cell lineCancer typeIC50 (μM)					
	A549	SK‐LU‐1	HCT‐15	PC3	L929	
	Lung carcinoma	Lung adenocarcinoma	Colorectal adenocarcinoma	Prostate adenocarcinoma	Nontumor	
VL1	7.0 ± 1.3	16.6 ± 1.6	3.14 ± 0.22	4.2 ± 0.5	41.6 ± 2.1^∗^	
VL2	1.07 ± 0.32	13.36 ± 0.87	3.47 ± 0.53	4.85 ± 0.56	55.9 ± 4.3^∗^	
VL3	4.68 ± 0.31	6.85 ± 0.98	2.62 ± 0.26	1.71 ± 0.31	7.6 ± 0.8	
Cisplatin	12.1 ± 1.7	3.23 ± 0.20	17.47 ± 0.38	7.61 ± 0.66	3.01 ± 0.55	

The results shown are relative values normalized with negative controls (72 h). Data are expressed as mean ± SEM to three independent experiments. ^∗^ *p* < 0.05 in comparison with cisplatin.

Cytotoxicity assays represent the initial step in evaluating new pharmaceutical molecules for potential in vivo application and subsequent clinical trials. In this context, the safety of complexes **VL**_**1**_ **–VL**_**3**_ was assessed in murine fibroblasts cell line (L929), a traditional model used to evaluate biocompatibility [47]. The results indicated that **VL**_**1**_ and **VL**_**2**_ exhibited significantly lower toxicity in normal cells compared to cisplatin, while **VL**_**3**_ demonstrated notable cytotoxicity against the L929 cells (Table 2). These findings underscore the therapeutic potential of **VL**_**1**_ and **VL**_**2**_, which combine high efficacy against cancer cells with reduced toxicity in normal healthy cells.

Vanadium, a nonplatinum group metal, has demonstrated antitumoral properties and is believed to play a role in biological systems, partly due to its ability to undergo facile redox reactions that enable interactions with biological molecules such as enzymes and receptors [3]. Vanadium complexes have been extensively studied for their potential anticancer activity, especially V (IV) and, to a lesser extent, V (V) [48–52]. Their anticancer activity has been demonstrated in a wide range of in vitro and in vivo models due to their redox properties and the ability to coordinate with various organic ligands, showing promising therapeutic applications. In contrast, there are a few reports involving V (III) complexes compared to their oxidized counterparts due to their low stability under physiological conditions [53, 54].

Vanadium complexes are recognized as labile systems that undergo rapid chemical conversions in aqueous biological environments. According to Crans, Costa‐Pessoa, and coworkers, these conversions include hydrolysis, ligand exchange, and changes in the oxidation state once the complexes are introduced into physiological media [55, 56]. Within this system, it is important to consider that the chemical species present at the low concentrations typically used in biological assays (μM range) may differ substantially from the parent complex initially administered. At physiological pH, many vanadium complexes are susceptible to hydrolysis and ligand exchange, leading to the formation of inorganic vanadate species such as monovanadate (H_2_VO_4_^−^) [57]. This species predominates near pH 7 and is well known to act as a transition‐state analog inhibitor of phosphatases due to its close structural similarity to phosphate [58–60].

In addition to hydrolytic processes, intracellular redox transformations occurred to further complicate vanadium speciation. Once inside the cell, V (V) species can be reduced to V (IV) by endogenous reductants such as glutathione or ascorbate, while the reverse oxidation of V (IV) back to V (V) may also occur depending on the cellular environment [61, 62]. As a result, identifying a single biologically active form is challenging. In this sense, vanadium coordination compounds are often considered pro‐drug systems, in which the coordinated ligands influence the initial transport, stability, and bioavailability of the metal center, whereas the observed biological activity frequently derives from the active species generated in situ following chemical transformation [63]. From this perspective, the biological results remain relevant, as the starting complex serves as the essential precursor from which the active vanadium species are produced.

The biological activity observed in our assays is consistent with a scenario in which the vanadium complexes undergo partial transformation in the biological medium. In buffered solutions at pH 7.4 and at micromolar concentrations, equilibria among coordinated complexes, partially hydrolyzed intermediates, and inorganic vanadate species are generated. Consequently, the measured cytotoxicity probably arises from a dynamic mixture of vanadium species rather than from a single, structurally well‐defined complex. This interpretation is further supported by the capacity of vanadium species to participate in redox processes, hydrolysis, and ligand‐exchange reactions under biological conditions [55]. In this sense, the **VL**_**2**_ and **VL**_**3**_ complexes should be identified as chemically defined precursors that modulate vanadium delivery and speciation, therefore indirectly determining the magnitude and profile of the observed biological response.

The synthesis and biological activity of V (III) complexed with amino acids such as cysteine or glycine have been reported in rat cancer models. For instance, [V^III^ (Hcys)_3_] ⋅2HCl, 5H_2_O] was found to prevent lung metastases in rats treated with 3,4‐benzopyrene. Two V (III) complexes containing N‐(2‐mercaptopropionyl)‐glycine and L‐cysteine were investigated for antitumor effects in rats [64]. The L‐cysteine complex demonstrated a significant antitumor activity against leiomyosarcomas at very low vanadium doses, whereas the N‐(2‐mercaptopropionyl) glycine complex showed weaker effects [65]. More recently, a combinatory therapy involving V (III)‐L‐cysteine complex with cyclophosphamide has been studied in mice bearing breast adenocarcinoma cells. The results demonstrated that the combination regimen decreased tumor mass and enhanced the survival of tumor‐bearing mice via the stimulation of reactive oxygen species (ROS) in tumor cells. The same complex was administered with cisplatin (CDDP), which enhanced CDDP‐induced antiproliferative activity in MCF‐7 and NCI‐H520 cancer cell lines [66].

To the best of our knowledge, no published studies have reported the anticancer activity of V(III) complexes with azole‐based ligands. Therefore, the **VL**_**1**_ **–VL**_**3**_ complexes represent novel compounds featuring V(III) centers with promising anticancer potential. The ligand framework is crucial in vanadium coordination, as complexation with organic ligands improves biological compatibility, reduces toxicity, and enhances activity against drug‐resistant cancer cells [3]. In this report, ligands 1,2,3‐benzotriazole‐pyridine and 1,2,4‐triazole‐pyridine were investigated, as triazole derivatives have recently been studied for their promising anticancer activity, particularly in combinatorial therapies, molecular hybrids with organic pharmacophores, and drug delivery systems [67–69]. Our results showed that ligands **L**_**1**_ **–L**_**3**_ alone do not exhibit significant cytotoxic effects against the cancer cell lines tested, but when forming complexes with V(III), their biological activity is significantly improved. Notably, 1,2,3‐benzotriazole derivatives have been explored as ligands in Ga(III) complexes displaying limited anticancer activity against HeLa, MCF‐7, and HT‐29 cell lines. In contrast, 1,2,3‐triazole and 1,2,4‐triazole anions have been employed as ligands in rhenium cluster complexes, where they facilitate DNA binding and contribute to enhanced cytotoxicity [70]. Broadly, triazoles have been extensively studied as ligands in metal complexes and as components of organic hybrids with potential antiproliferative activity [71]. These findings highlight the promise of azole‐based ligands in cancer research and further investigate their coordination with V (III).

Considering the present findings, it is hypothesized that both the metal center and the organic ligand contribute to the DNA‐binding mechanism of the complexes. The pyridine moiety likely imparts electron‐withdrawing properties, which may enhance electrostatic interactions with the negatively charged DNA backbone. Simultaneously, the benzotriazole or triazole unit may promote intercalation between the DNA base pairs and donate electron density, thereby reinforcing the ligand’s binding affinity [72, 73]. The V (III) center is capable of coordinating with nitrogen or oxygen donor atoms within the DNA bases or phosphate backbone, potentially facilitating strong binding, inducing conformational changes, or disrupting the double helix. Previous studies have reported DNA cleavage activity by V(III) complexes, including bis(maltolato)V(III)‐polypyridyl compounds and dimeric species such as [(VCl(Phen)_2_)_2_O]^2+^ and [(VCl(Bpy)_2_)_2_O]^2+^, which exhibited enhanced plasmid DNA cleavage in the presence of ligands such as dipyrido[3,2‐*d*:2′,3′‐*f*]quinoxaline(dpq), dipyrido[3,2‐*a*:2′,3′‐*c*]phenazine (dppz) and phenanthroline (Phen). Importantly, an effective anticancer agent should selectively target malignant cells while minimizing cytotoxic effects on normal, healthy tissues [74, 75].

Hence, a desirable anticancer agent should preferentially target tumor cells while sparing healthy tissue, we calculated the SI for each compound, defined as the ratio of IC_50_ in normal cells (L929) to IC_50_ in cancer cells (Table 3). A higher SI value indicates greater tumor specificity. **VL**_**2**_ exhibited the highest selectivity among all compounds tested, with particularly favorable SI values in A549 (52.2), HCT‐15 (16.1), and PC3 (11.5). **VL**_**1**_ also showed a promising SI in HCT‐15 cells (13.3). Weerapreeyakul et al. suggest a lower threshold (SI ≥ 3) for identifying promising anticancer candidates [76]. It is important to emphasize that assessing a sample’s anticancer activity solely in malignant cell lines or animal models, without determining its SI, is an unreliable predictor for further (clinical) development [77]. **VL**_**1**_ and **VL**_**2**_ meet or exceed the threshold in several cancer cell lines by this criterion. Furthermore, although the **VL**_**3**_ complex exhibited a superior anticancer activity compared to **VL**_**1**_ and **VL**_**2**_, it displayed a lower SI. Nonetheless, **VL**_**3**_ demonstrated a higher SI than cisplatin, which is reported to exhibit SI values of ≤ 0.7. Cisplatin remains one of the earliest and most effective metal‐based chemotherapeutic agents, widely employed in the treatment of various malignancies, including cancers of the bladder, head and neck, lungs, ovaries, and testes. However, its clinical application is significantly limited by severe dose‐dependent toxicities, such as nephrotoxicity, ototoxicity, neurotoxicity, and myelosuppression. These adverse effects are primarily attributed to the poor selectivity of platinum‐based chemotherapeutics toward cancer cells over healthy tissue.

**TABLE 3:** Selectivity index (SI) of cancer cell lines treated with V (III) complex **VL**_**1**_ **–VL**_**3**_ for 72 h.

Compound	Selectivity index (SI)			
	A549	SK‐LU	HCT15	PC3
VL_1_	5.9	2.5	13.3	9.9
VL_2_	52.2	4.2	16.1	11.5
VL_3_	1.6	1.1	2.9	4.4
Cisplatin	0.25	0.93	0.17	0.40

SI = (IC_50_ control cells/IC_50_ cancer cells).

In general, the series of complexes **VL**_**1**_ **–VL**_**3**_ exhibited higher SIs than cisplatin. In addition to their selectivity, the complexes demonstrated notable chemical stability over time (Figures 6 and 7). Unlike cisplatin, which is light‐sensitive and requires protection from light to maintain its stability and therapeutic efficacy, the **VL**_**1**_ **–VL**_**3**_ complexes did not require special storage conditions. A time‐dependent decline in cisplatin’s potency was observed, with its IC_50_ value increasing by approximately 1.5‐fold per week (data not shown). In contrast, the **VL**_**1**_ **–VL**_**3**_ complexes maintained consistent inhibitory concentrations in solution over time, which agrees with the stability assay results discussed earlier in this report (Section 2.5).

### 3.7. Flow Cytometry Analysis

**FIGURE 9:**
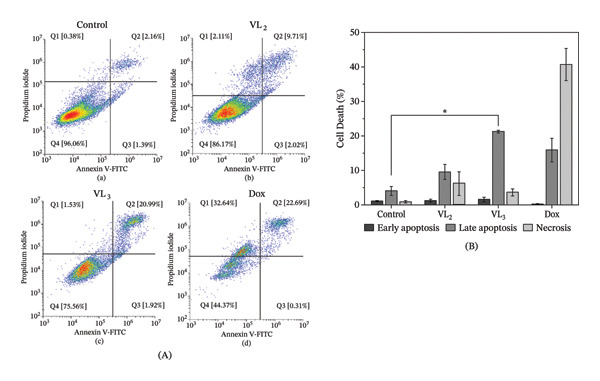
The apoptotic effects of vanadium (III) complexes: (a) control; (b) **VL**_**2**_ (12 μM)**;** (c) **VL**_**3**_ (20 μM); (d) positive control for apoptosis (doxorubicin 10 μM) in A549 cell line. Cells were stained by Annexin V‐FITC and PI and analyzed by flow cytometry. (A) Representative flow cytometry analysis of cell population in necrosis (Q1); late apoptosis (Q2); early apoptosis (Q3). (B) Quantification analysis by treatment. Data are expressed as mean ± SEM to three independent experiments. ∗*p* < 0.05 compared with the negative control.

To investigate whether the reduction in A549 cell viability induced by the vanadium(III) complexes was mediated through apoptotic pathways, cell death mechanisms were evaluated using Annexin V/PI staining and analyzed by flow cytometry (Figure 9).

Analysis of apoptosis showed that none of the treatments significantly altered early apoptosis compared with the control group. In contrast, a significant increase in late apoptosis was observed following treatment with **VL**_**3**_ (20 μM). Treatment with **VL**_**2**_ (12 μM) did not produce a significant change in apoptosis relative to the control. As expected, the positive control doxorubicin (10 μM) induced a marked apoptotic response, confirming the sensitivity and validity of the experimental model.

Doxorubicin also induced a pronounced increase in necrosis compared with the control, consistent with its role as a cytotoxic reference compound [78]. In contrast, neither of the vanadium(III) complexes evaluated induced a significant increase in necrotic cell death, with values remaining comparable to the control group. Collectively, these results indicate that **VL**_**2**_ exhibits greater selectivity in cytotoxicity assays toward cancer cells, whereas **VL**_**3**_ shows higher overall cytotoxic potency but lower selectivity toward the healthy cell line. Consistent with these observations, **VL**_**3**_ promotes late apoptosis without triggering necrosis, while **VL**_**2**_ does not significantly affect apoptotic or necrotic pathways under the conditions tested. These findings are further supported further by the docking studies.

### 3.8. Molecular Docking of Vanadium Complexes With DNA Target

The binding energy analysis of vanadium complexes (Table 4) relative to cisplatin provides insights into their potential anticancer activity; cisplatin, a standard anticancer agent, has a binding energy of −6.3 kcal/mol, serving as a benchmark for DNA affinity. Among the vanadium complexes, **VL**_**1**_ shows the least favorable binding energy (−4.78 kcal/mol), indicating a weaker DNA interaction. In contrast, **VL**_**2**_ exhibits a binding energy of −6.66 kcal/mol, suggesting a slightly stronger affinity for DNA than cisplatin. The **VL**_**3**_ complex has the highest binding energy at −7.07 kcal/mol, implying the strongest DNA interaction among the complexes tested. This trend in binding energies (**VL**_**3**_ > **VL**_**2**_ > cisplatin > **VL**_**1**_) underscores the impact of structural differences on DNA‐binding affinity.

**TABLE 4:** Binding energies of V (III) complexes and cisplatin with DNA (PDB: 1AIO).

	Cisplatin	VL_1_	VL_2_	VL_3_
Binding energy (kcal/mol)	−6.3	−4.78	−6.66	−7.07

**FIGURE 10:**
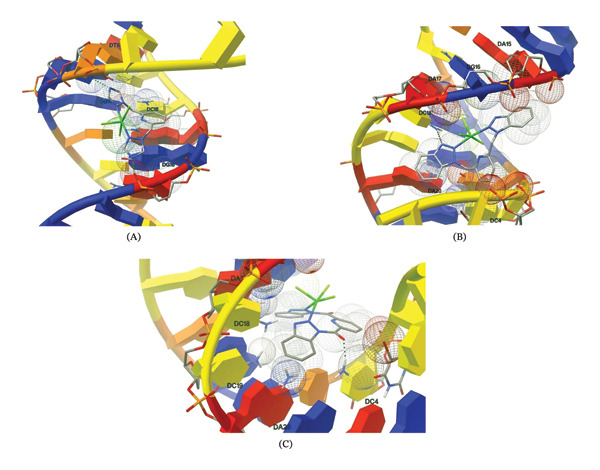
Interaction diagrams of the vanadium complexes with DNA, highlighting key contacts, hydrogen bonds are shown in black, with vanadium atoms in green, nitrogen in blue, and chlorine in yellow green, green dashed lines indicate the unfavorable contacts. The complexes are labeled as follows: (A) **VL**_**1**_, (B) **VL**_**2**_, and (C) **VL**_**3**_.

In terms of specific interactions, the weaker binding of **VL**_**1**_ can be attributed to the electrostatic repulsion between its 1,2,4‐triazole group and the phosphate backbone of DNA (Figure 10). On the other hand, **VL**_**2**_’s improved affinity can be linked to its methyl group, which enhances conformational flexibility and allows more favorable positioning within the DNA‐binding site. Additionally, the aromatic rings in **VL**_**2**_ engage in hydrophobic interactions with DNA bases and form hydrogen bonds with cytosine‐6. The strong binding of **VL**_**3**_ is due in part to its carbonyl linker (C=O), which restricts flexibility, stabilizing the **fac** conformation and promoting a precise DNA fit. This conformation facilitates hydrophobic interactions with DNA and enables a hydrogen bond via the carbonyl group of the vanadium complex.

### 3.9. Ethidium Bromide Displacement Assay

Molecular docking studies align with cytotoxicity assay results, revealing that the **VL**_**3**_ complex exhibits the most substantial growth inhibition in cancer cells. However, it also affects normal cells, indicating limited selectivity. While **VL**_**3**_ demonstrates potent antiproliferative activity, its cytotoxicity diminishes its potential as a therapeutic agent, a common drawback among current metallodrugs. In contrast, the **VL**_**2**_ complex shows comparable anticancer efficacy and strong DNA‐binding affinity, yet with significantly reduced toxicity toward normal cells.

To confirm the DNA interaction of **VL**_**2**_ as predicted by molecular docking, an ethidium bromide (EtBr) displacement assay was conducted. EtBr is a well‐known intercalating DNA binder due to its planar aromatic structure, which enhances DNA fluorescence by up to 20 times upon binding. This property makes EtBr a widely used probe in fluorescence displacement assays to evaluate the DNA‐binding ability of new compounds.

**FIGURE 11:**
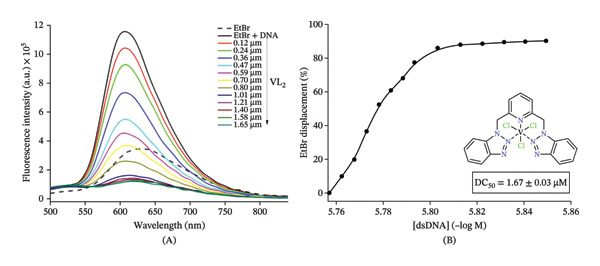
Ethidium bromide (EtBr) displacement assay: (A) emission spectrum of the EtBr‐DNA adduct with increasing concentrations (0.12–1.65 μM) of complex **VL**_**2**_. (B) Dose–response curve for the titration of dsDNA with complex **VL**_**2**_ and DC_50_ value.

In this study, the extent of fluorescence quenching in the dsDNA–EtBr system was used to assess the interaction between the **VL**_**2**_ complex and DNA. As shown in Figure 11A, fluorescence emission at 610 nm progressively decreased with increasing concentrations of **VL**_**2**_, indicating that EtBr molecules were being displaced from their DNA‐binding sites.

The mean displacement concentration (DC_50_) for **VL**_**2**_ was determined to be 1.62 μM (Figure 11B), suggesting that **VL**_**2**_ interacts with DNA through intercalation or partial intercalation. This binding mode is likely stabilized by π–π stacking interactions between the aromatic rings of the benzotriazole or pyridine moieties in **VL**_**2**_ and the DNA base pairs. Such interactions can induce structural alterations in the DNA helix, including elongation, increased rigidity, and partial unwinding.

The displacement of EtBr by vanadium compounds has been reported in multiple studies, suggesting a possible intercalative binding mechanism [79–81]. The displacement of ethidium bromide (EtBr) by vanadium compounds has been reported in several studies, indicating that these species can interact with DNA and compete with intercalated EtBr for binding sites. While this behavior is often associated with an intercalative binding mode, the interaction may involve either the intact vanadium complex or vanadium‐containing species formed through ligand exchange or hydrolysis under experimental conditions.

A study on a dinuclear dioxide vanadium(V) complex also demonstrated DNA interaction and displacement of EtBr from CT‐DNA–EtBr adducts. The authors proposed that the mode of action may involve electron transfer in the excited state or disruption of the DNA’s secondary structure [82].

While the metal center in coordination complexes plays a critical role in their biological activity, the ligands significantly influence their function. For instance, platinum‐based complexes that bind to DNA through intercalation are well‐known for their anticancer properties. However, studies have shown that the ligands attached to these complexes strongly affect their cytotoxicity, suggesting that their biological activity involves more than just DNA binding it is likely to include additional intracellular interactions [83, 84].

Based on the results of the EtBr fluorescence displacement experiments, we conclude that **VL**_**2**_ interacts with dsDNA through intercalation or partial intercalation mechanisms. These findings are in strong agreement with the molecular docking results discussed earlier. In conclusion, the combined biological, stability, and in silico studies identify the **VL**_**2**_ complex as the most promising candidate among the evaluated V(III) complexes, highlighting its notable anticancer activity and high selectivity.

### 3.10. LogP Study for Ligands and Complexes

The calculated lipophilicity values (logP) of the vanadium complexes show a clear correlation with their experimental cytotoxic activity (Table S3). **VL**_**2**_ and **VL**_**3**_ exhibit significantly higher logP values (2.87 and 2.15, respectively) compared to **VL**_**1**_, indicating a more pronounced hydrophobic character. Increased lipophilicity is a critical factor in anticancer drug activity, as it enhances membrane permeability and facilitates intracellular accumulation, thereby improving access to biological targets such as DNA. Consistent with this, **VL**_**2**_ and **VL**_**3**_ demonstrated the most potent cytotoxic effects across multiple cancer cell lines, with **VL**_**2**_ showing exceptional activity against A549 cells (IC_50_ = 1.07 μM) and VL3 exhibiting strong activity in PC3 (IC_50_ = 1.71 μM) and HCT‐15 cells (IC_50_ = 2.62 μM). In contrast, **VL**_**1**_, which has a markedly lower logP and thus a more hydrophilic character, showed comparatively weaker cytotoxic activity. The higher lipophilicity of **VL**_**2**_ and **VL**_**3**_ likely enhances hydrophobic interactions with DNA bases and promotes cellular uptake, contributing to their improved biological efficacy. These findings highlight the importance of ligand design in modulating lipophilicity and demonstrate that an optimal balance of hydrophobicity is essential for maximizing the anticancer activity of vanadium complexes.

## 4. Conclusions

In this study, a series of vanadium (III) complexes bearing azole‐based ligands (VL_1_–VL_3_) were synthesized, structurally characterized, and evaluated for their cytotoxic activity against a panel of cancer cell lines. The crystal structure of the VL3D complex was elucidated by single‐crystal x‐ray diffraction. All complexes exhibited a significant antiproliferative activity, particularly against lung (A549, SK‐LU‐1), colorectal (HCT‐15), and prostate (PC3) cancer cells, with VL_2_ and VL_3_ displaying the highest cytotoxic potency. These results suggest that both the vanadium center and the nature of the organic ligand play key roles in the biological activity and DNA‐binding properties of the complexes.

*In silico* DNA‐binding studies revealed that VL_3_ exhibited the strongest affinity for DNA, surpassing that of cisplatin, likely due to the structural rigidity conferred by its carbonyl linker, which enables optimal hydrophobic interactions and hydrogen bonding. VL_2_ also demonstrated strong DNA binding, potentially enhanced by the flexibility introduced by its methyl substituent; this interaction was further supported by the ethidium bromide displacement assay. In contrast, VL_1_ showed a weaker DNA interaction, possibly due to repulsive effects associated with its 1,2,4‐triazole moiety.

Mechanistic studies provided further insight into the biological effects of these complexes. Apoptosis analysis in A549 cells revealed that VL_3_ selectively promoted late‐stage apoptosis without inducing necrosis, consistent with its higher cytotoxic potency but limited selectivity toward normal cells. In contrast, VL_2_ did not significantly affect apoptotic or necrotic pathways under the conditions tested, suggesting that its antiproliferative activity may involve alternative or combined mechanisms in a more controlled mode of action. These findings align with the cytotoxicity and selectivity data and support distinct cellular responses elicited by VL_2_ and VL_3_.

Regarding safety, VL_1_ and VL_2_ were found to be less toxic than cisplatin in murine fibroblasts and exhibited a higher SI for cancer cells, suggesting a better therapeutic index. VL_3_, however, showed limited selectivity, indicating that its efficacy may be accompanied by unwanted toxicity. Also, the VL_2_ complex is the most promising as an anticancer agent due to its significant tumor growth inhibition and reduced toxicity in normal cells.

The robust activity observed in several lines (e.g., PC‐3, HCT‐15, A549, and SK‐LU‐1) supports the broad‐spectrum potential of VL_1_–VL_3_, while the minimal response in MCF‐7 highlights the limitation of extrapolating its efficacy to hormone‐responsive breast cancers. These findings provide a solid foundation for the future development of V(III)‐based complexes with enhanced DNA‐targeting properties and improved therapeutic profiles.

## Funding

This study was funded by Universidad de Los Andes under Projects Nos. INV‐2023‐162‐2718, INV‐2021–118–2225, INV‐2022–137–2385, and INV‐2025‐213‐3347. This work was supported by the Foundation for the Promotion of Research and Technology of the Bank of the Republic, Project Code 5.261.

## Conflicts of Interest

The authors declare no conflicts of interest.

## Supporting Information

Additional supporting information can be found online in the Supporting Information section.

## Supporting information


**Supporting Information** Supporting 1. Additional supporting information can be found online in the Supporting Information section. (*Supporting Information*). Supplementary 2. Supporting information associated with this article includes: Supporting 3. Figure S1. 400 MHz ^1^H NMR (DMSO‐*d*_6_) spectrum of *L*_1_. Supporting 4. Figure S2. 400 MHz ^1^H NMR (DMSO‐*d*_6_) spectrum of *L*_2_. Supporting 5. Figure S3. 400 MHz ^1^H NMR (DMSO‐*d*_6_) spectrum of *L*_3_. Supporting 6. Figure S4. FT‐IR spectra of *L*_1_. Supporting 7. Figure S5. FT‐IR spectra of *L*_2_. Supporting 8. Figure S6. FT‐IR spectra of *L*_3_. Supporting 9. Figure S7. FT‐IR spectra of VL_1_. Supporting 10. Figure S8. FT‐IR spectra of VL_2_. Supporting 11. Figure S9. FT‐IR spectra of VL_3_. Supporting 12. Figure S10. Raman spectra of VCl_3_ in the range of 100–400 cm^−1^. Supporting 13. Figure S11. Raman spectra of VL_1_ in the range of 100–400 cm^−1^. Supporting 14. Figure S12. Raman spectra of VL_2_ in the range of 100–400 cm^−1^. Supporting 15. Figure S13. Raman spectra of VL_3_ in the range of 100–400 cm^−1^. Supporting 16. Figure S14. Mass spectrum of VL_1_. Supporting 17. Figure S15. Mass spectrum of VL_2_. Supporting 18. Figure S16. Mass spectrum of VL_3_. Supporting 19. Figure S17. UV–vis spectrum in DMSO of *L*_1_ at 2.5 × 10^−4^ M. Supporting 20. Figure S18. UV–vis spectrum in DMSO of *L*_2_ at 2.5 × 10^−4^ M. Supporting 21. Figure S19. UV–vis spectrum in DMSO of *L*_3_ at 2.5 × 10^−4^ M. Supporting 22. Figure S20. UV–vis spectrum in DMSO of VCl_3_ in the range of 240–340 nm at 2.5 × 10^−4^ M. Supporting 23. Figure S21. UV–vis spectrum in DMSO of VCl_3_ in the range of 420–550 nm at 1 × 10^−3^ M. Supporting 24. Figure S22. UV–vis spectrum in DMSO of VCl_3_ in the range of 590–800 nm at 1 × 10^−3^ M. Supporting 25. Figure S23. UV–vis spectrum in DMSO of VL_1_ in the range of 400–600 nm at 1 × 10^−3^ M. Supporting 26. Figure S24. UV–vis spectrum in DMSO of VL_1_ in the range of 620–760 nm at 1 × 10^−3^ M. Supporting 27. Figure S25 UV–vis spectrum in DMSO of VL_2_ in the range of 550–900 nm at 1 × 10^−3^ M. Supporting 28. Figure S26. UV–vis spectrum in DMSO of VL_3_ in the range of 580–900 nm at 1 × 10^−3^ M. Supporting 29. Figure S27. UV–vis spectrum of the stability in DMSO of VL_1_ − VL_3_ at 2.5 × 10^−4^ M (D: day). Supporting 30. Figure S28. UV–vis spectrum of the stability in H_2_O:DMSO (95:5) of VL_1_ − VL_3_ (D: day). Supporting 31. Figure S29. TGA and derivative thermogravimetric (DTG) of VL_1_ in a nitrogen atmosphere. Supporting 32. Figure S30. TGA and derivative thermogravimetric (DTG) of VL_2_ in a nitrogen atmosphere. Supporting 33. Figure S31. TGA and derivative thermogravimetric (DTG) of VL_3_ in a nitrogen atmosphere. Supporting 34. Figure S32. Dose–response curves for vanadium (III) complex *V* *L*_1_ − *V* *L*_3_. Supporting 35. Figure S33. Cyclization process in the presence of water. A) Initial interaction involving the dissociation of water and subsequent formation and release of HCl. B) Nucleophilic attack by the hydroxyl group resulting in the cyclization and protonation of the triazole ligand. Distances in Armstrongs. Supporting 36. Figure S34. Second cyclization process in the presence of water (distances in Armstrongs). Supporting 37. Table S1. Crystal data, data collection, and refinement information of VL_3_D. Supporting 38. Table S2. Cytotoxic screening of ligands *L*_1_ − *L*_3_ and their vanadium (III) complexes VL_1_ − VL_3_ against cancer cell lines. Supporting 39. Table S3. Log *p* values for ligands and complexes.

## Data Availability

Data available are in the article supporting material.
