# Supplementary material for: Synthesis, Characterization, and Anticancer Activity of Vanadium (III) Complexes With Pyridyl–Triazole Ligands
Source: Bioinorg Chem Appl. 2026 Jun 15;2026:2823543. doi: 10.1155/bca/2823543 (PMC13269649; doi:10.1155/bca/2823543)
Supplement: Supplementary file 1 — Supporting Information Supporting 1. Additional supporting information can be found online in the Supporting Information section. (Supporting Information). Supplementary 2. Supporting information associated with this article includes: Supporting 3. Figure S1. 400 MHz 1H NMR (DMSO‐d 6) spectrum of L 1. Supporting 4. Figure S2. 400 MHz 1H NMR (DMSO‐d 6) spectrum of L 2. Supporting 5. Figure S3. 400 MHz 1H NMR (DMSO‐d 6) spectrum of L 3. Supporting 6. Figure S4. FT‐IR spectra of L 1. Supporting 7. Figure S5. FT‐IR spectra of L 2. Supporting 8. Figure S6. FT‐IR spectra of L 3. Supporting 9. Figure S7. FT‐IR spectra of VL1. Supporting 10. Figure S8. FT‐IR spectra of VL2. Supporting 11. Figure S9. FT‐IR spectra of VL3. Supporting 12. Figure S10. Raman spectra of VCl3 in the range of 100–400 cm−1. Supporting 13. Figure S11. Raman spectra of VL1 in the range of 100–400 cm−1. Supporting 14. Figure S12. Raman spectra of VL2 in the range of 100–400 cm−1. Supporting 15. Figure S13. Raman spectra of VL3 in the range of 100–400 cm−1. Supporting 16. Figure S14. Mass spectrum of VL1. Supporting 17. Figure S15. Mass spectrum of VL2. Supporting 18. Figure S16. Mass spectrum of VL3. Supporting 19. Figure S17. UV–vis spectrum in DMSO of L 1 at 2.5 × 10^−4^ M. Supporting 20. Figure S18. UV–vis spectrum in DMSO of L 2 at 2.5 × 10^−4^ M. Supporting 21. Figure S19. UV–vis spectrum in DMSO of L 3 at 2.5 × 10^−4^ M. Supporting 22. Figure S20. UV–vis spectrum in DMSO of VCl3 in the range of 240–340 nm at 2.5 × 10^−4^ M. Supporting 23. Figure S21. UV–vis spectrum in DMSO of VCl3 in the range of 420–550 nm at 1 × 10^−3^ M. Supporting 24. Figure S22. UV–vis spectrum in DMSO of VCl3 in the range of 590–800 nm at 1 × 10^−3^ M. Supporting 25. Figure S23. UV–vis spectrum in DMSO of VL1 in the range of 400–600 nm at 1 × 10^−3^ M. Supporting 26. Figure S24. UV–vis spectrum in DMSO of VL1 in the range of 620–760 nm at 1 × 10^−3^ M. Supporting 27. Figure S25 UV–vis spectrum in DMSO of VL2 in the range of 550–900 nm at [file BCA-2026-2823543-s001.pdf]

Supplementary Material

Synthesis, Characterization and Anticancer Activity of Vanadium(III) Complexes with Pyridyl–Triazole Ligands

Yair Alvarez-Ricardo*[1], Deissy N. Jaramillo[2], Adrian Orjuela Rocha[3], Elizabeth Jiménez-Díaz[2], Jorge Alí-Torres[4], Mario A. Macías[5], Antonino Arenaza-Corona[6], David Morales-Morales[6], John J. Hurtado*[1]

[1]*Research Group in Inorganic Chemistry, Catalysis and Bioinorganic Chemistry, Department of Chemistry, Universidad de los Andes, 1st Avenue No. 18A-12, Bogota 111711, Colombia*

[2]*Applied Biochemistry Research Group (GIBA), Department of Chemistry, Universidad de los Andes, 1st Avenue No. 18A-12, Bogota 111711, Colombia*

[3]*Institute of Scientific Research and High Technology Services (INDICASAT AIP), Panama City, Panama.*

[4]*Department of Chemistry, Universidad Nacional de Colombia – Bogota Campus, Colombia*

[5]*Crystallography and Chemistry of Materials, CrisQuimMat, Department of Chemistry, Universidad de los Andes, 1st Avenue No. 18A-12, Bogota 111711, Colombia*

[6]*Institute of Chemistry, Universidad Nacional Autónoma de México, Outer Circuit, University City, Mexico City, C.P. 04510, Mexico*

Corresponding authors: jj.hurtado@uniandes.edu.co, y.alvarezricardo@uniandes.edu.co; Phone: +57-1-3394949 (ext. 3468), ORCID: https://orcid.org/0000-0002-0511-9719, https://orcid.org/0000-0001-7001-3358

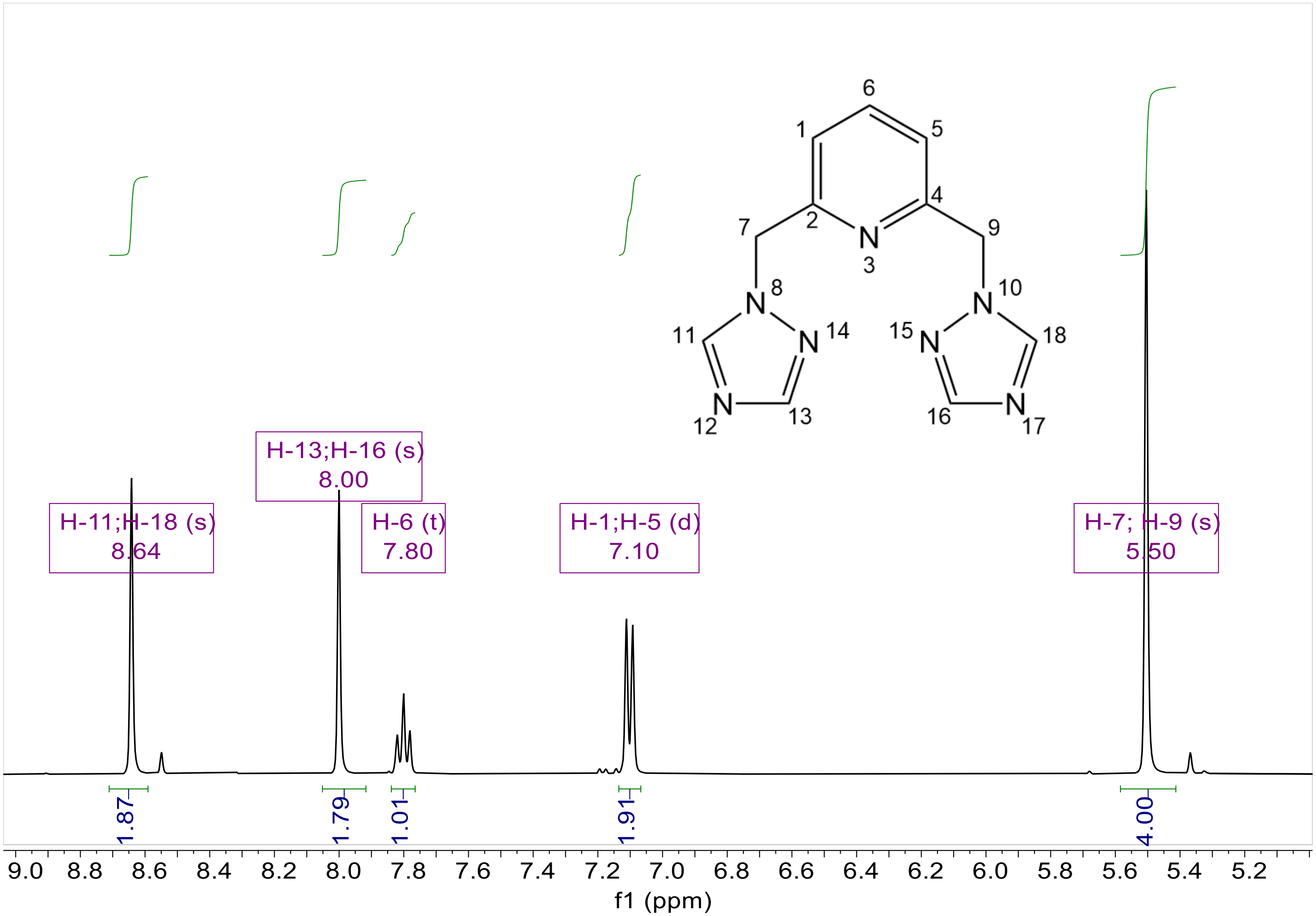

Figure S1. 400 MHz ^{1}H NMR (DMSO-d_6) spectrum of $\mathbf{L_1}$

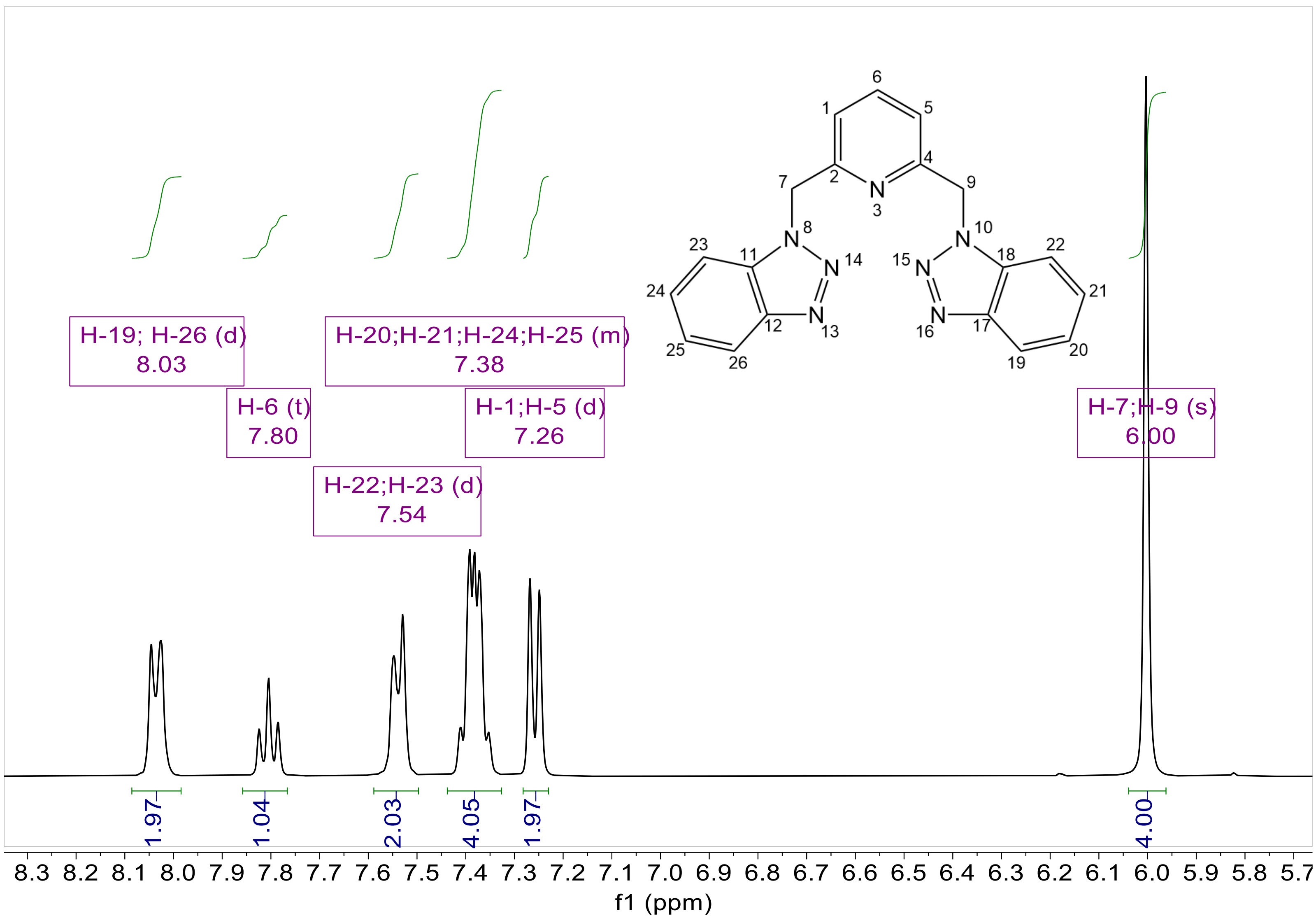

Figure S2. 400 MHz ^{1}H NMR (DMSO-d_6) spectrum of $\mathbf{L_2}$

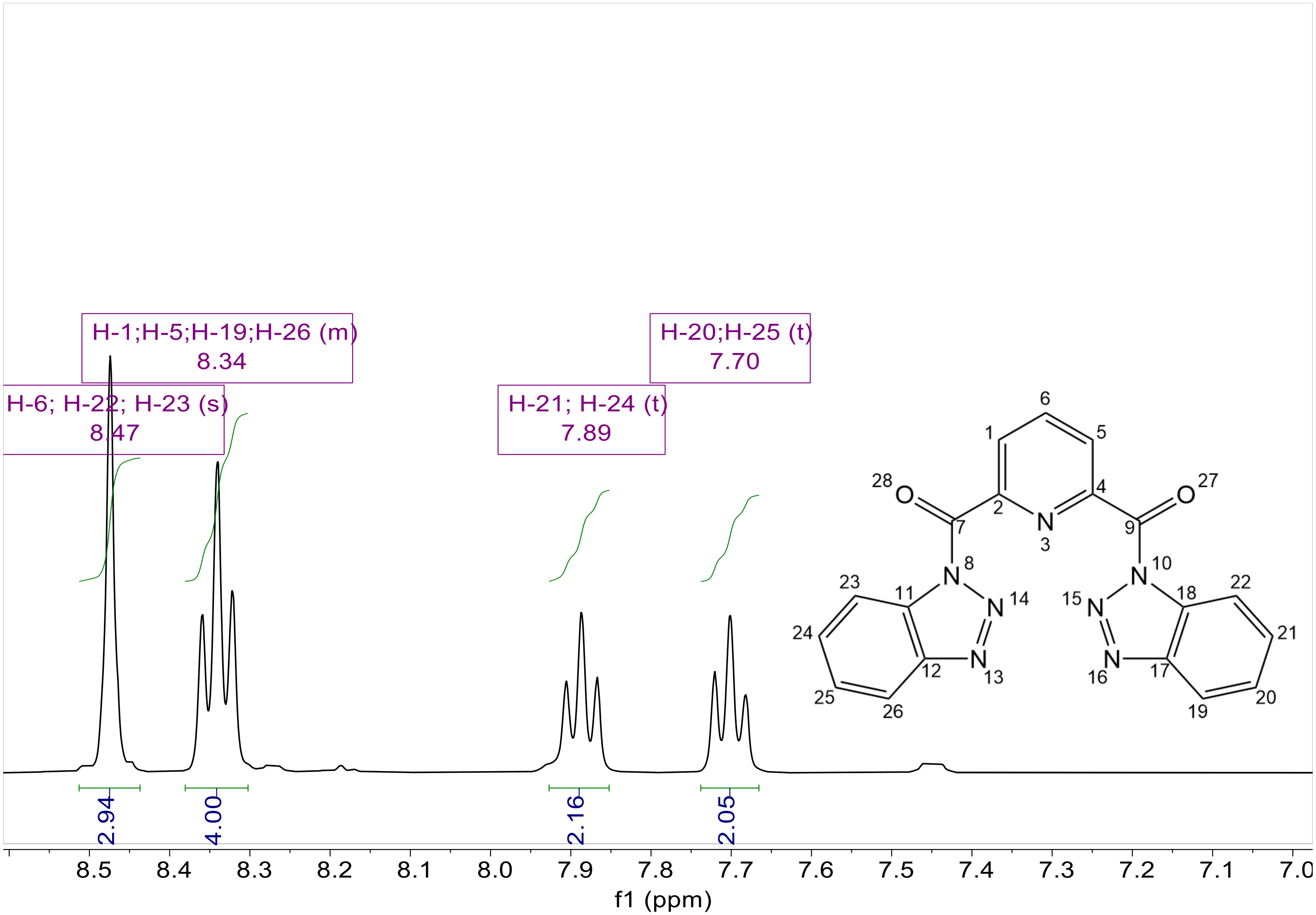

Figure S3. 400 MHz ^{1}H NMR (DMSO-d_6) spectrum of $\mathbf{L_3}$

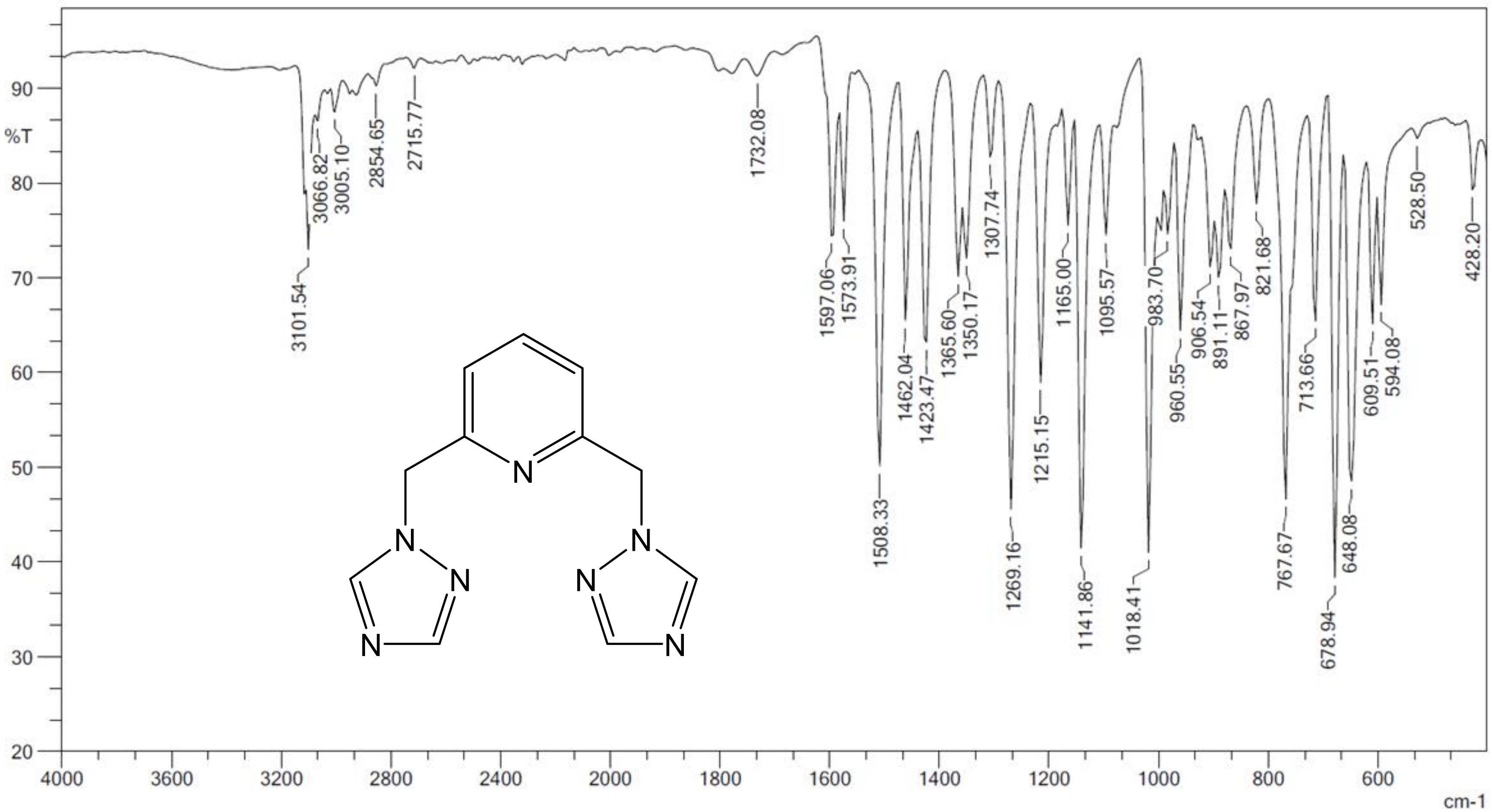

Figure S4. FT-IR spectra of $\mathbf{L_1}$

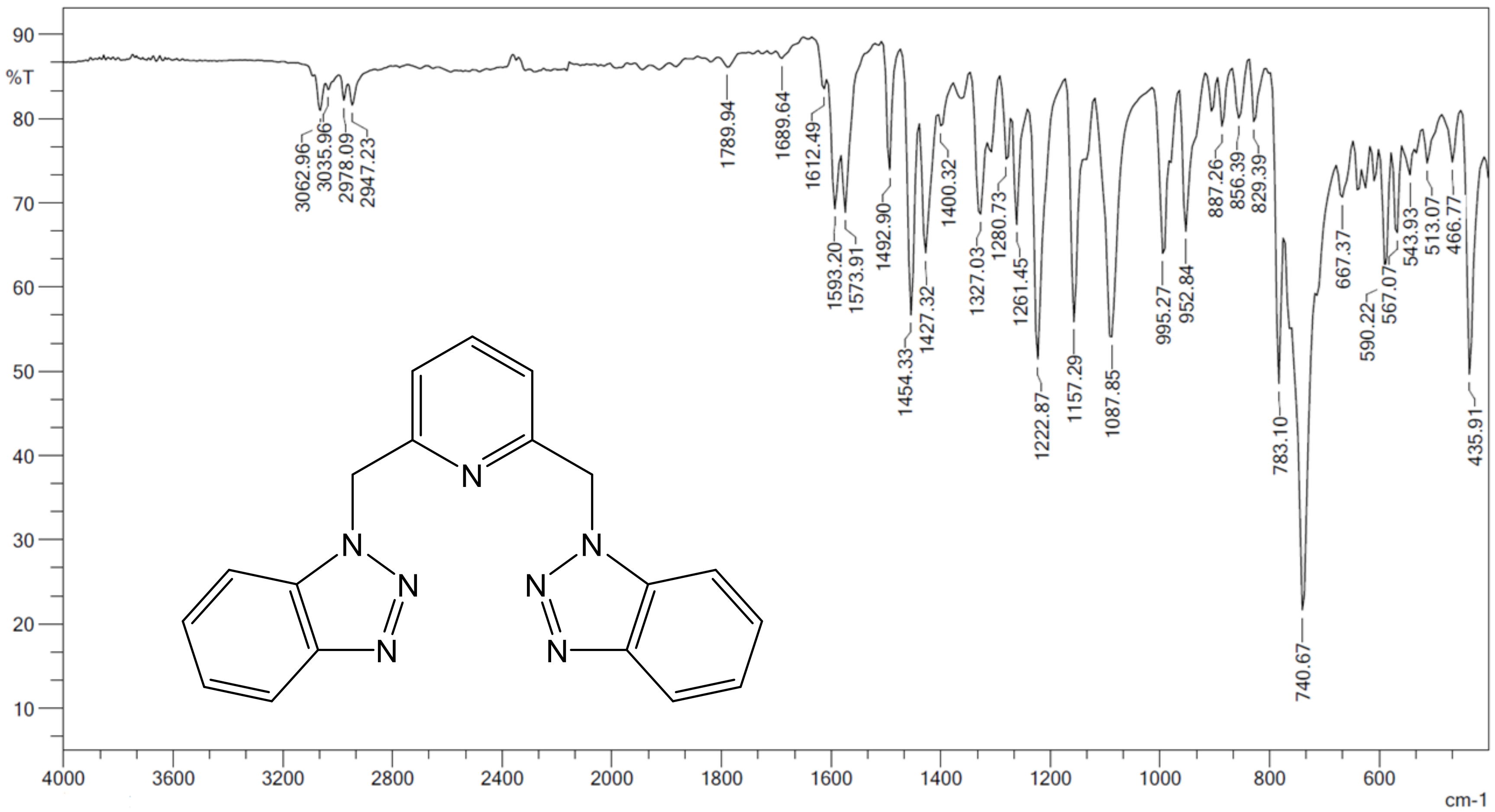

Figure S5. FT-IR spectra of $\mathbf{L_2}$

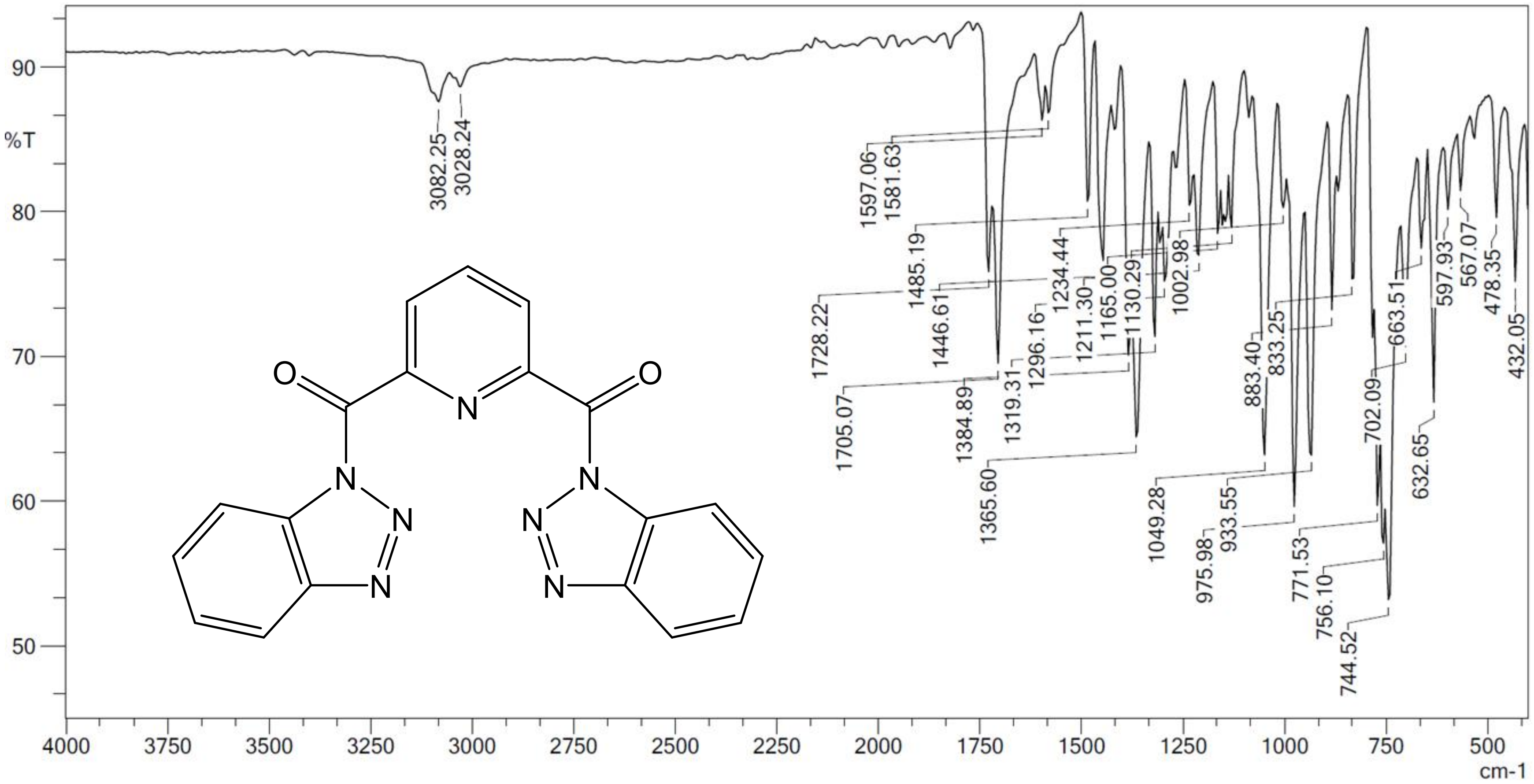

Figure S6. FT-IR spectra of **L$_3$**

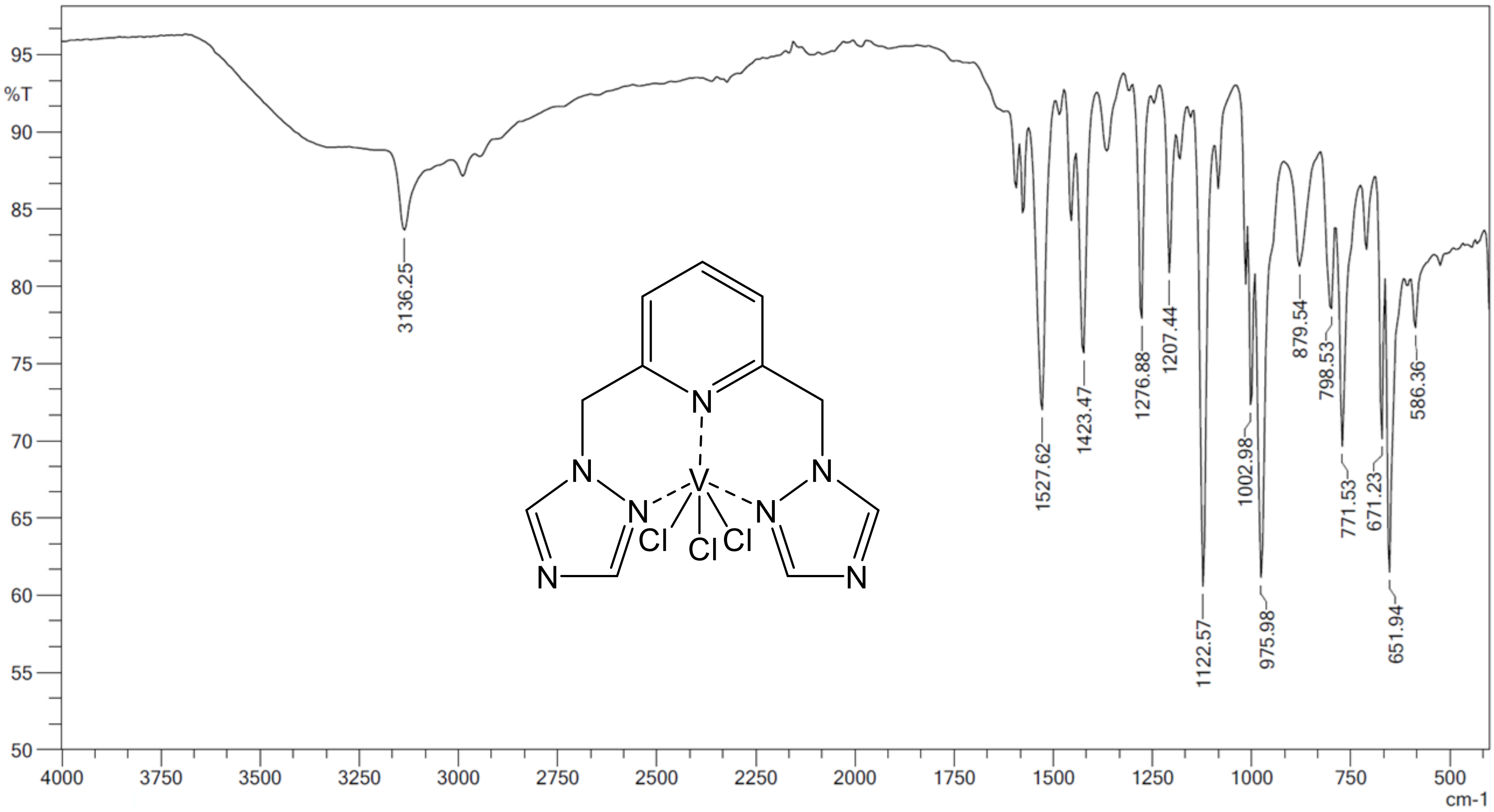

Figure S7. FT-IR spectra of $\mathbf{VL_1}$

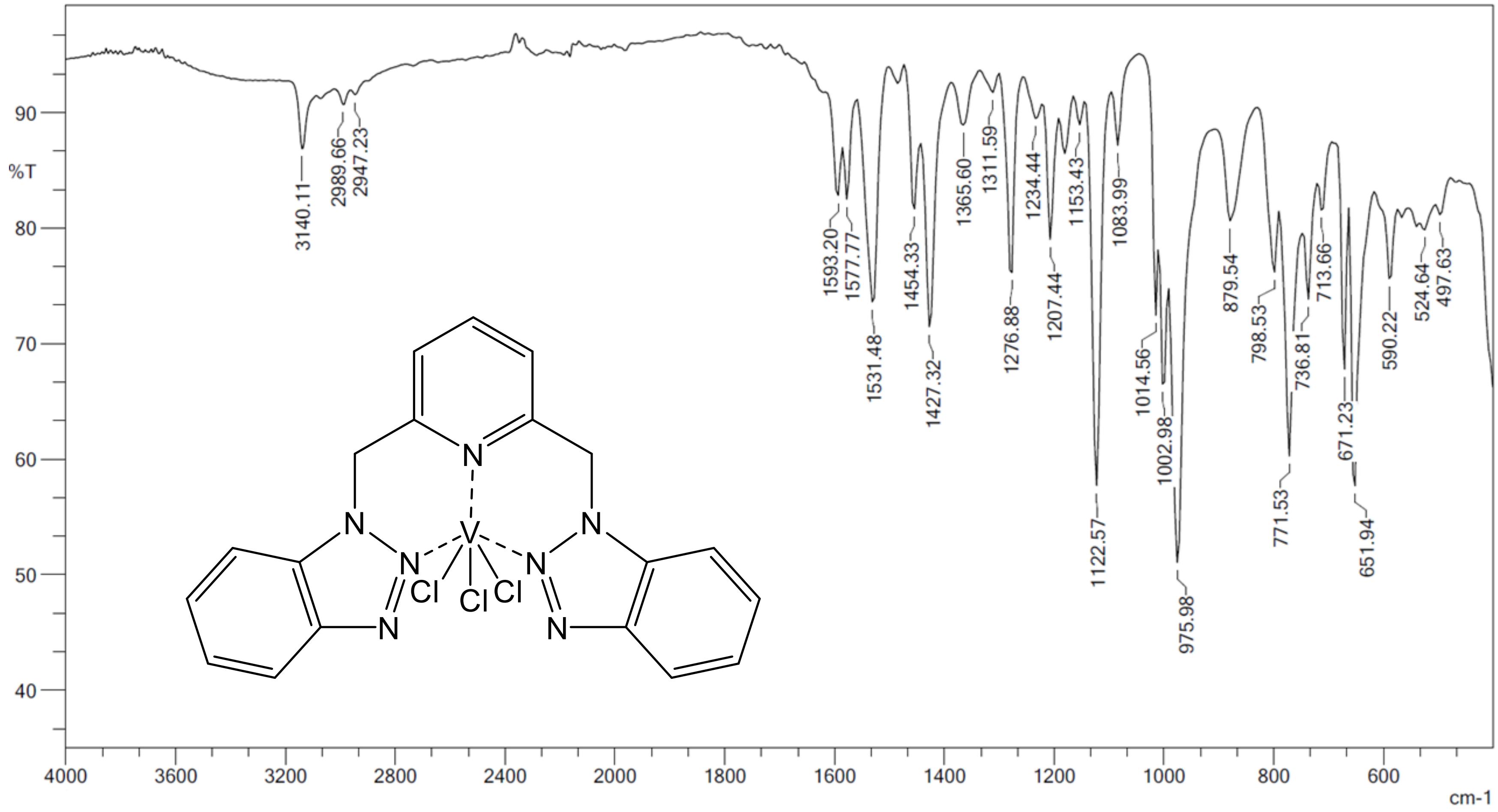

Figure S8. FT-IR spectra of **VL_2**

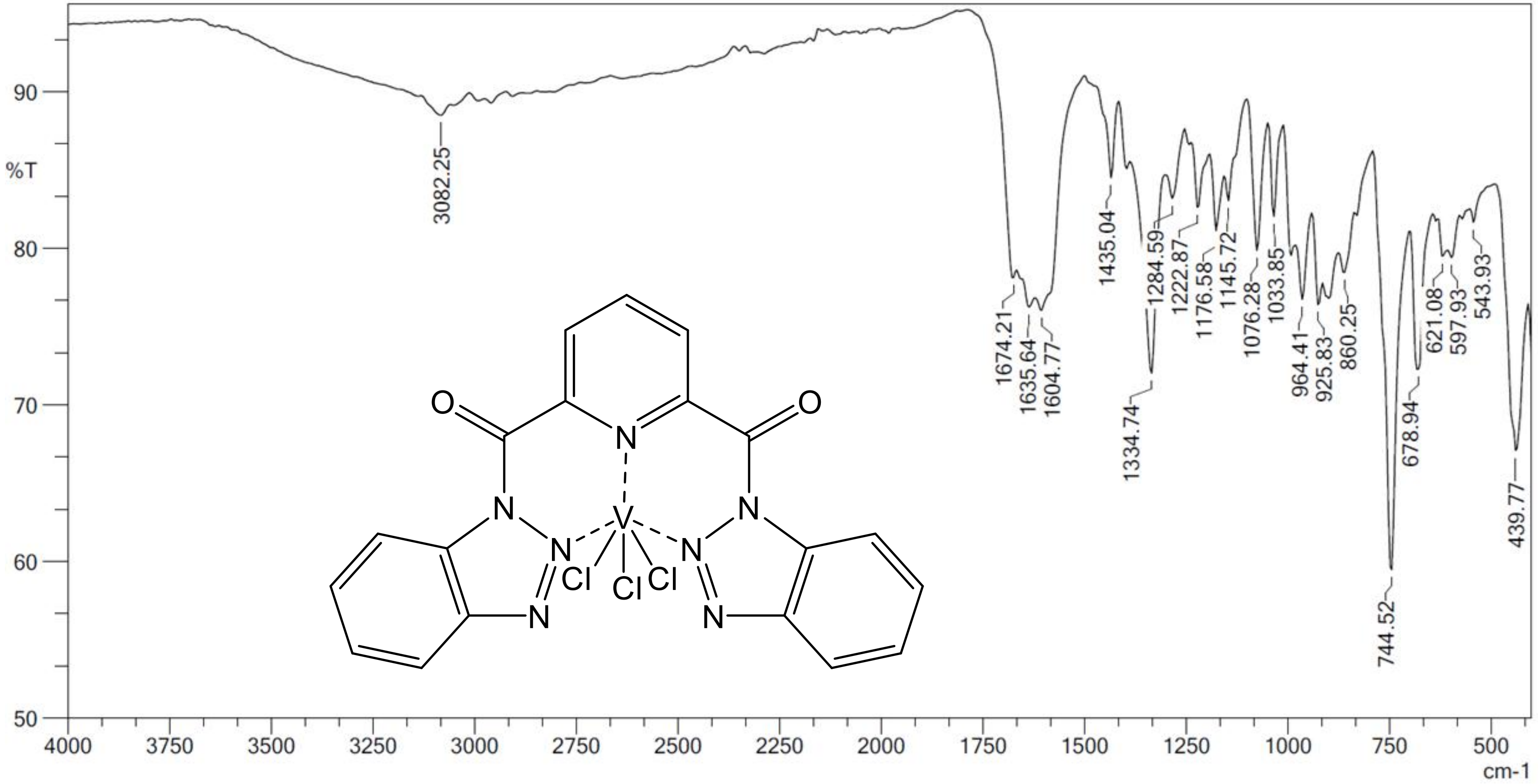

Figure S9. FT-IR spectra of $\mathbf{VL_3}$

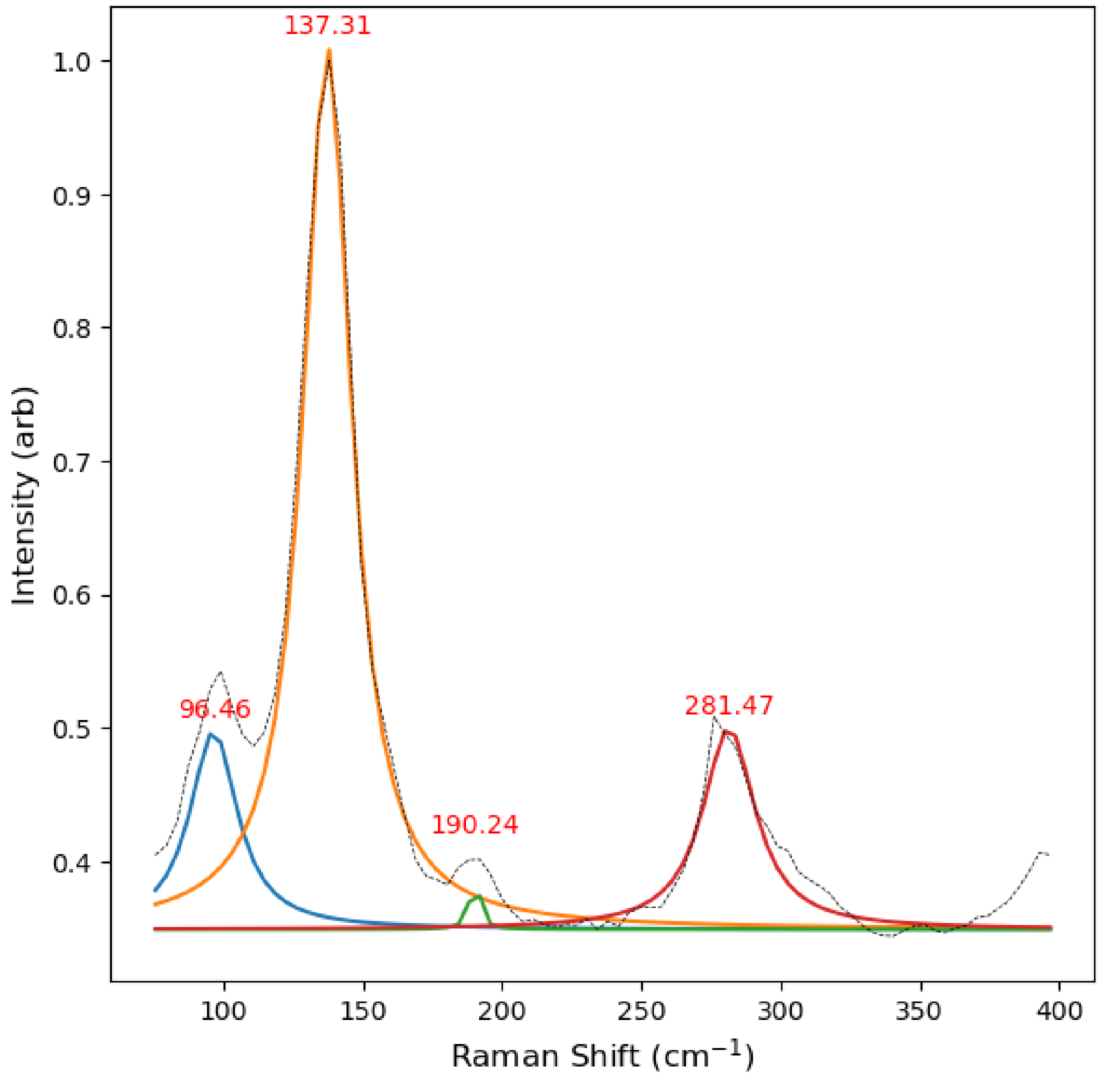

Figure S10. Raman spectra of $\mathbf{VCl_3}$ in the range of 100 – 400 cm^{-1}

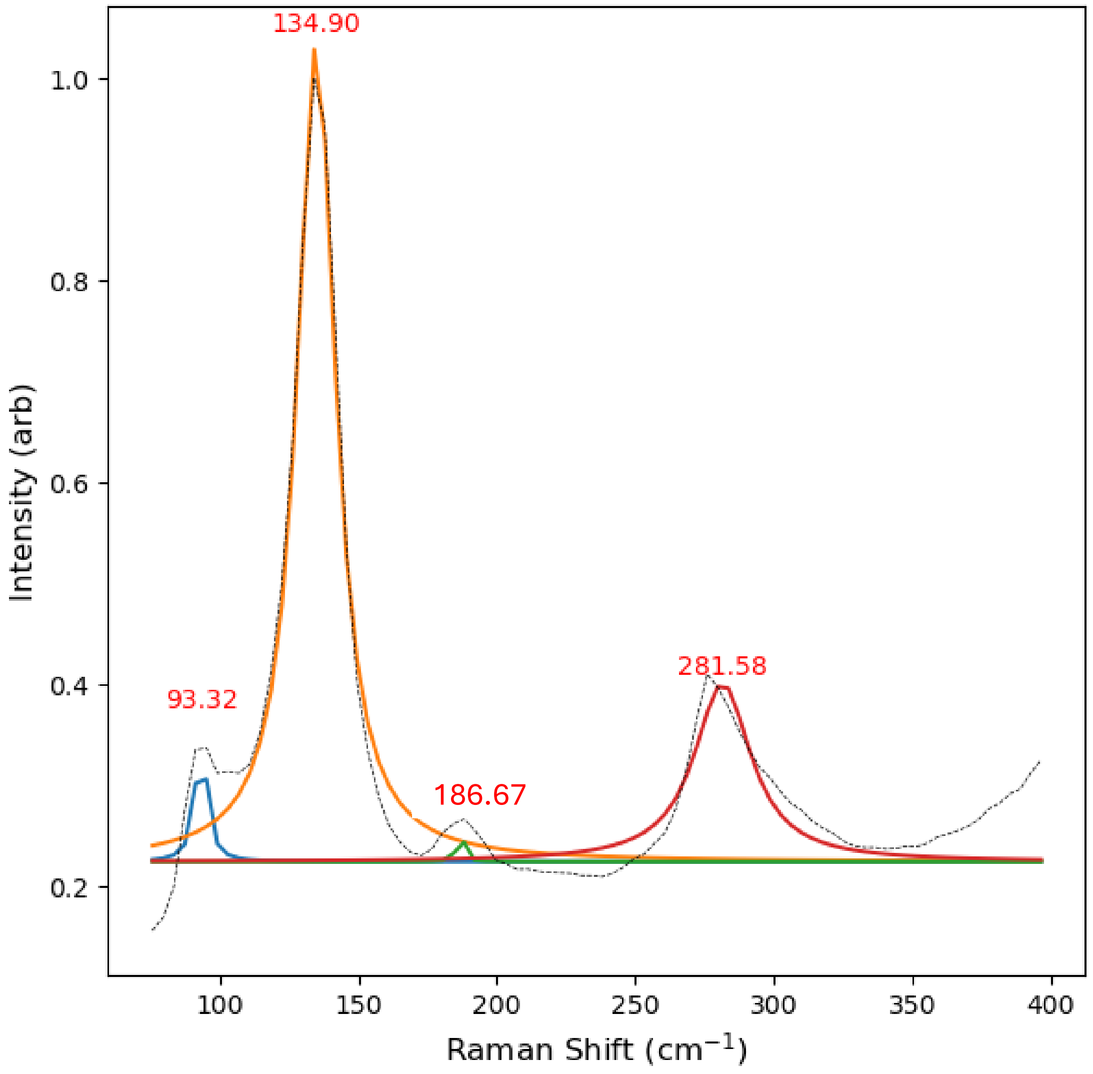

Figure S11. Raman spectra of **VL$_1$** in the range of 100 – 400 cm^{-1}

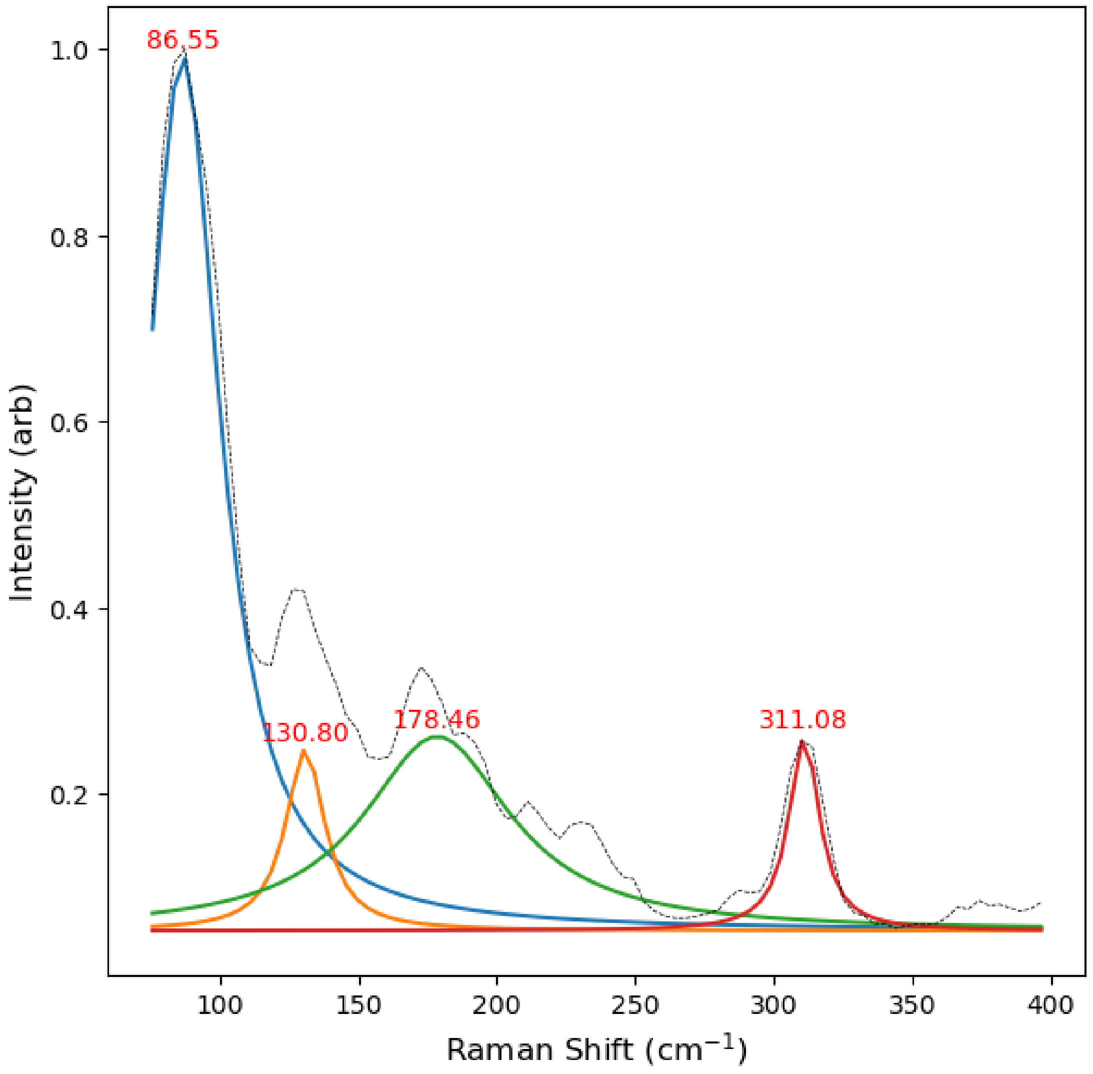

Figure S12. Raman spectra of **VL_2** in the range of 100 – 400 cm^{-1}

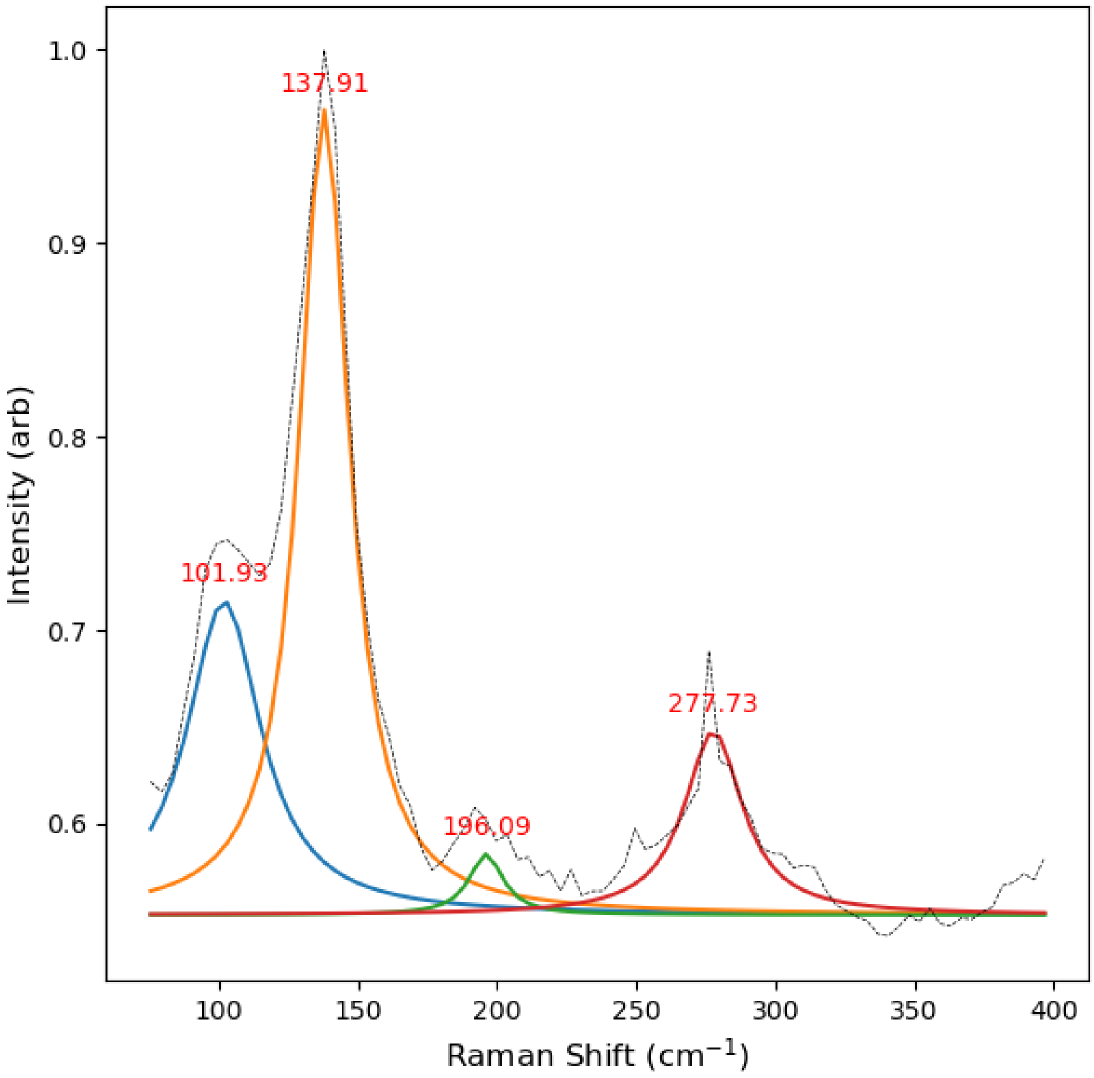

Figure S13. Raman spectra of **VL_3** in the range of 100 – 400 cm^{-1}

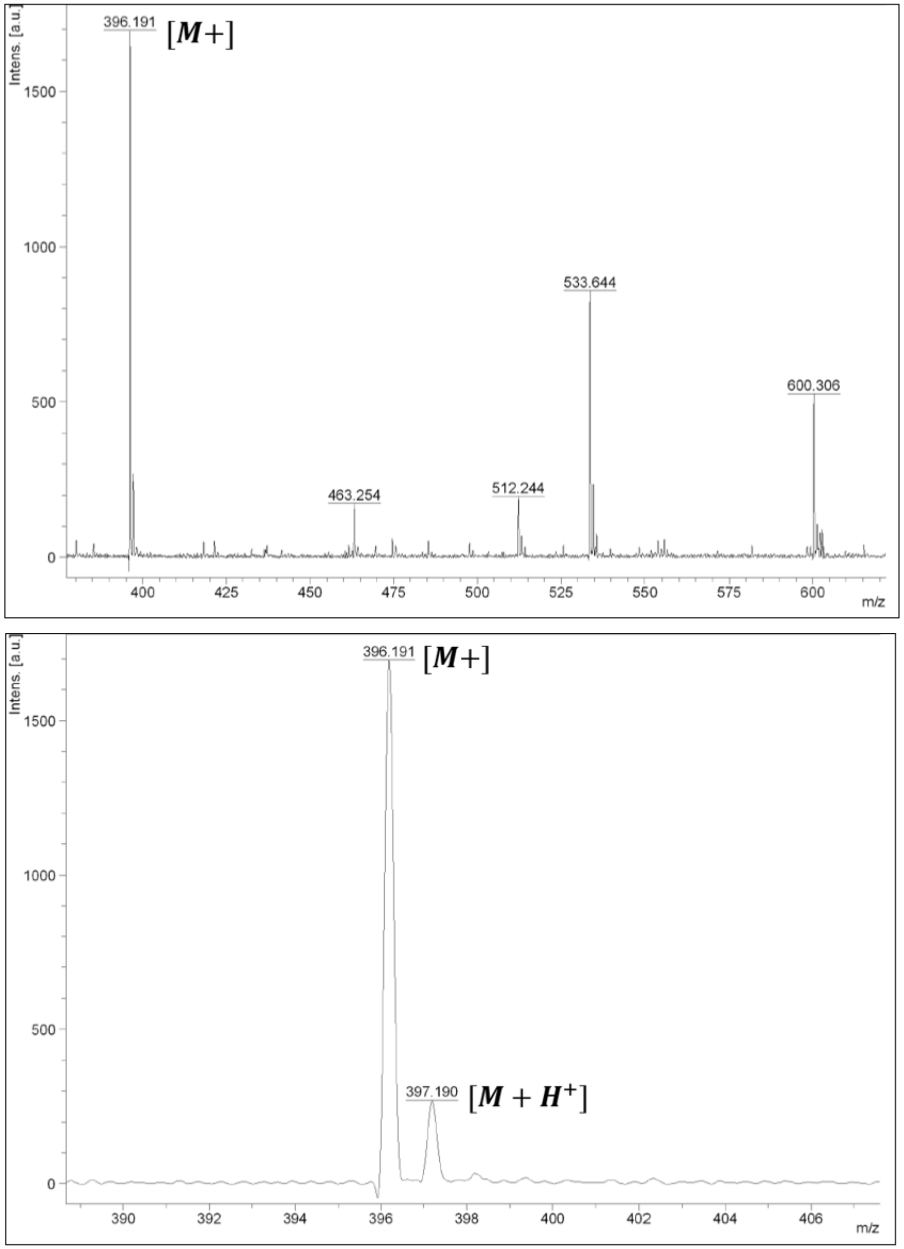

Figure S14. Mass spectrum of $\mathbf{VL_1}$

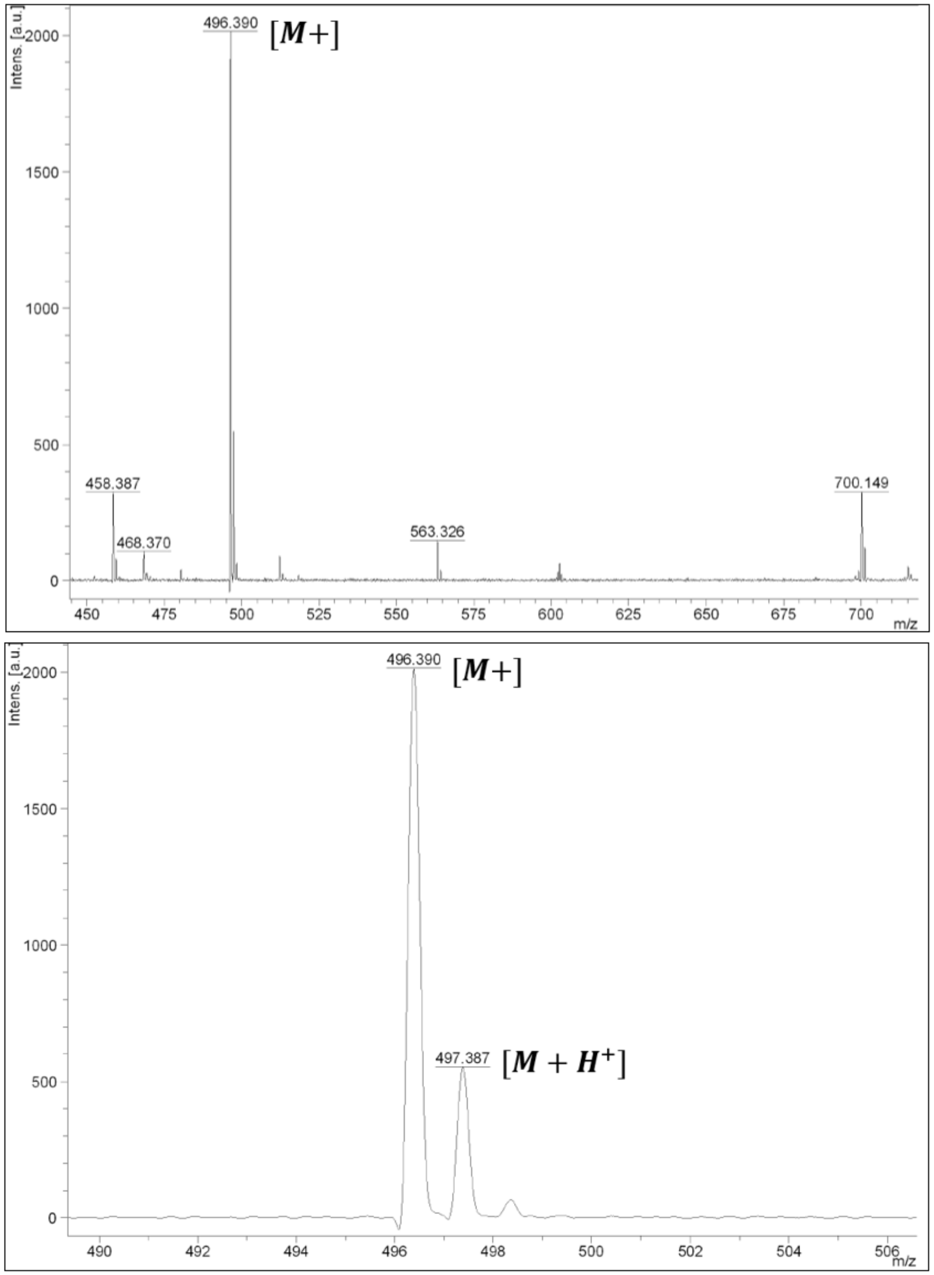

Figure S15. Mass spectrum of **VL$_2$**

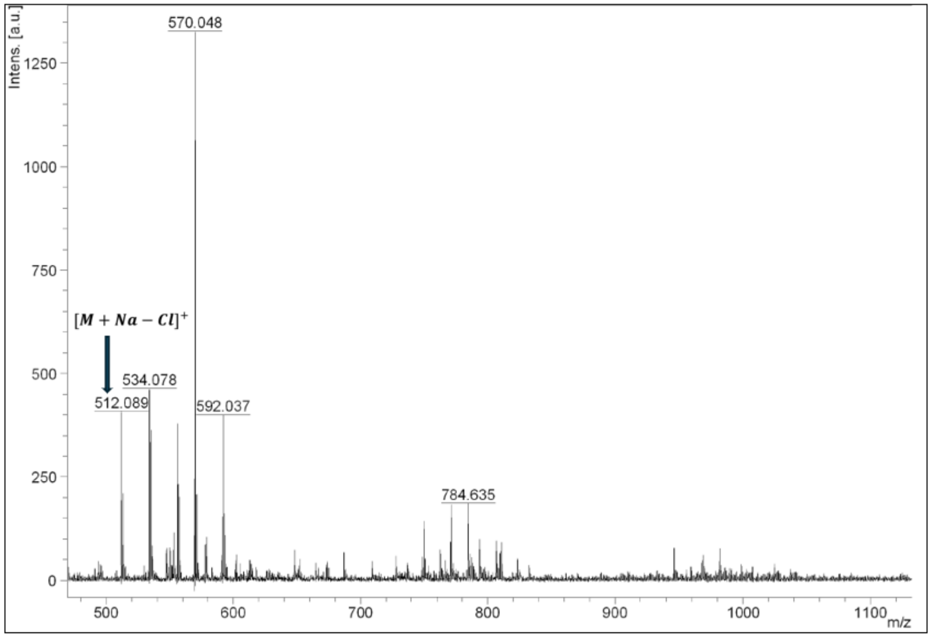

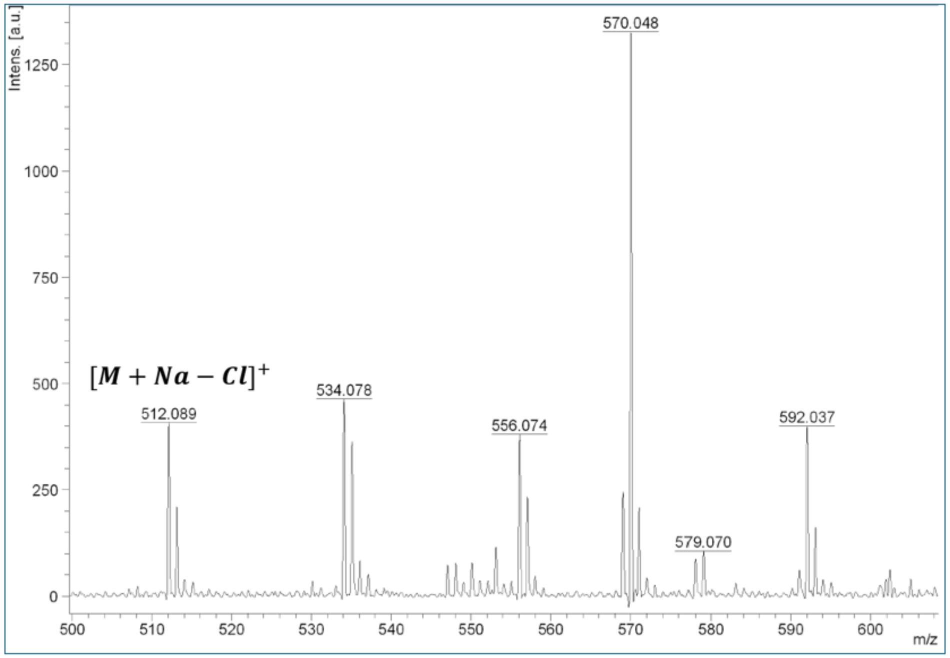

Figure S16. Mass spectrum of **VL_3**

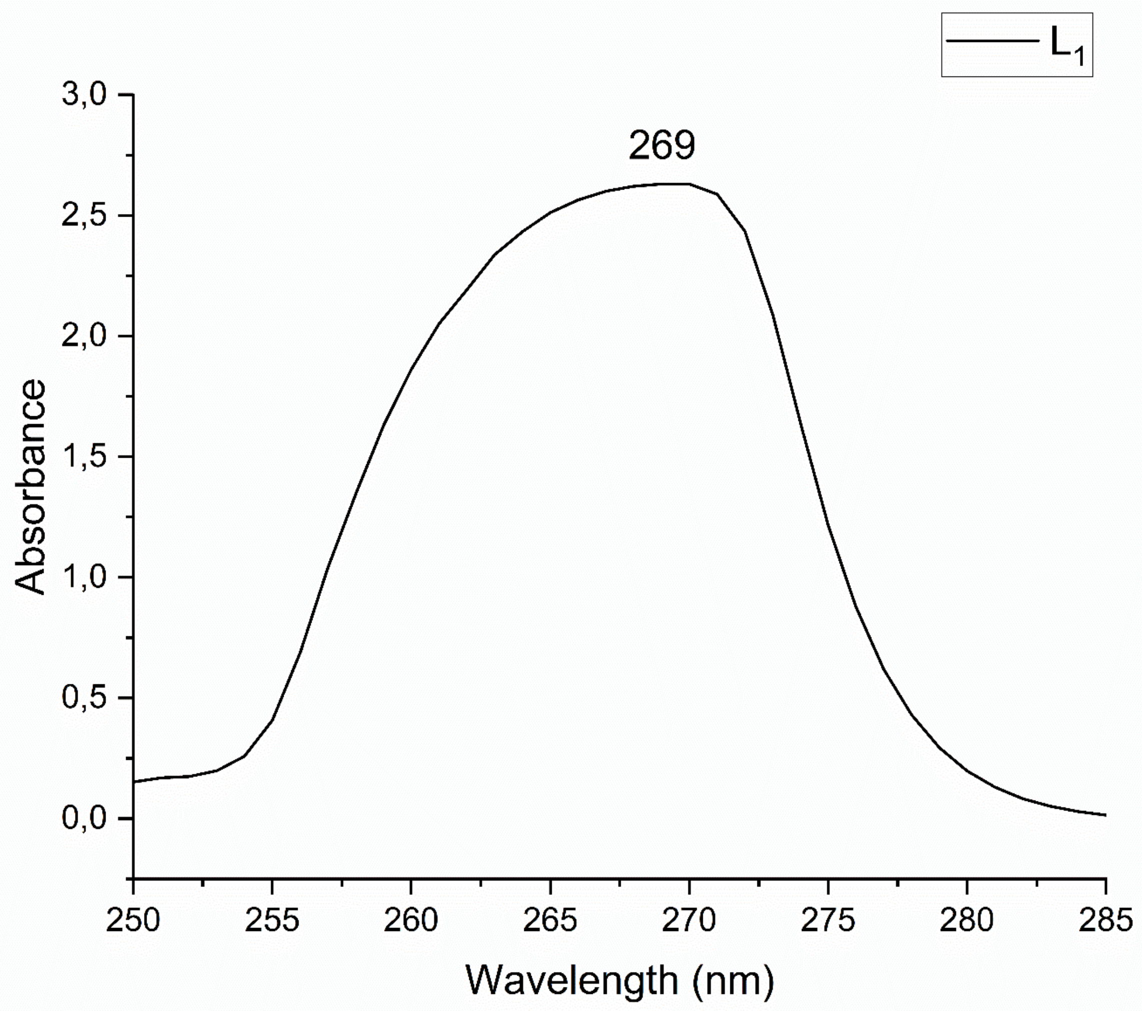

Figure S17. UV-VIS spectrum in DMSO of $\mathbf{L_1}$ at 2.5×10^{-4} M

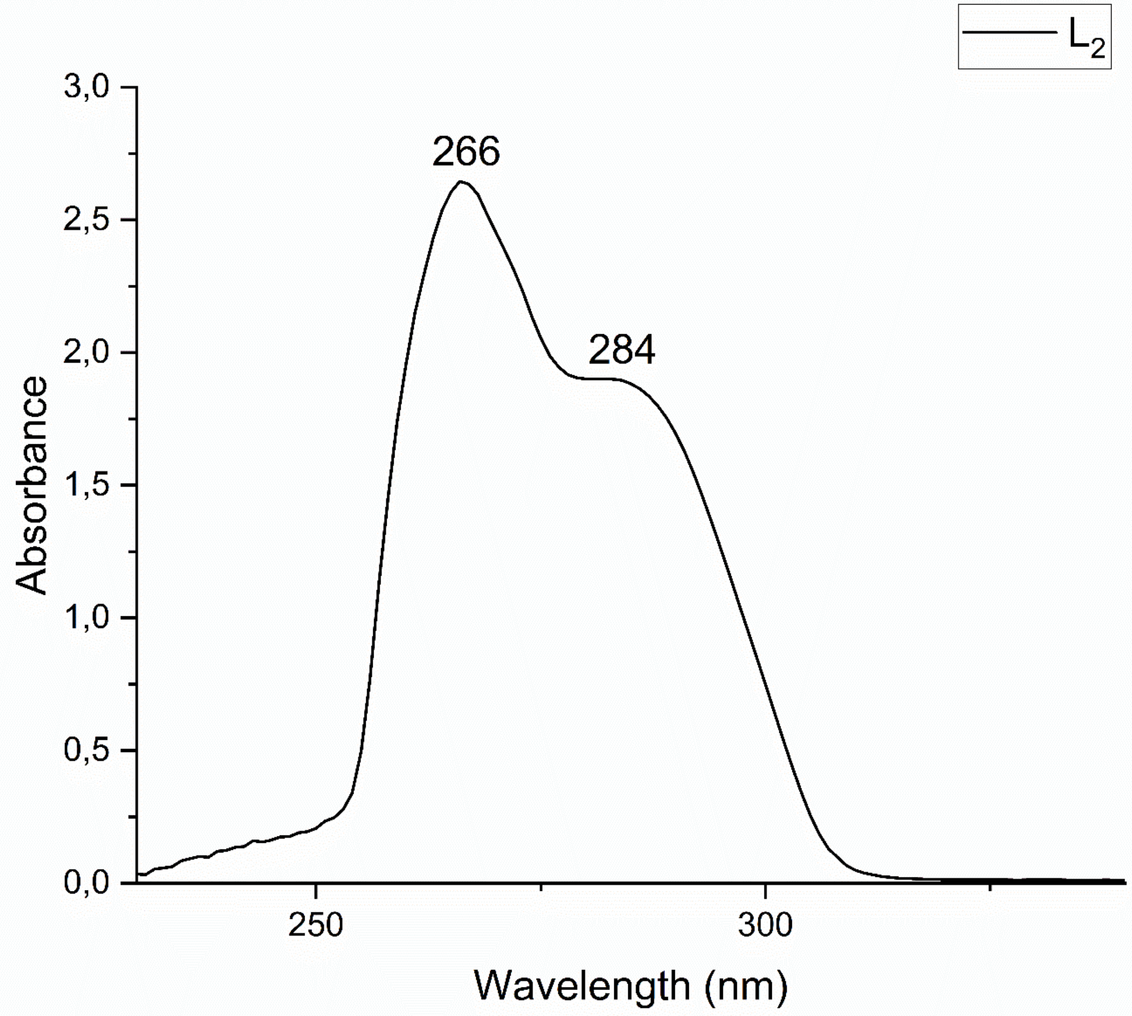

Figure S18. UV-VIS spectrum in DMSO of $\mathbf{L_2}$ at $2.5x10^{-4}$ M

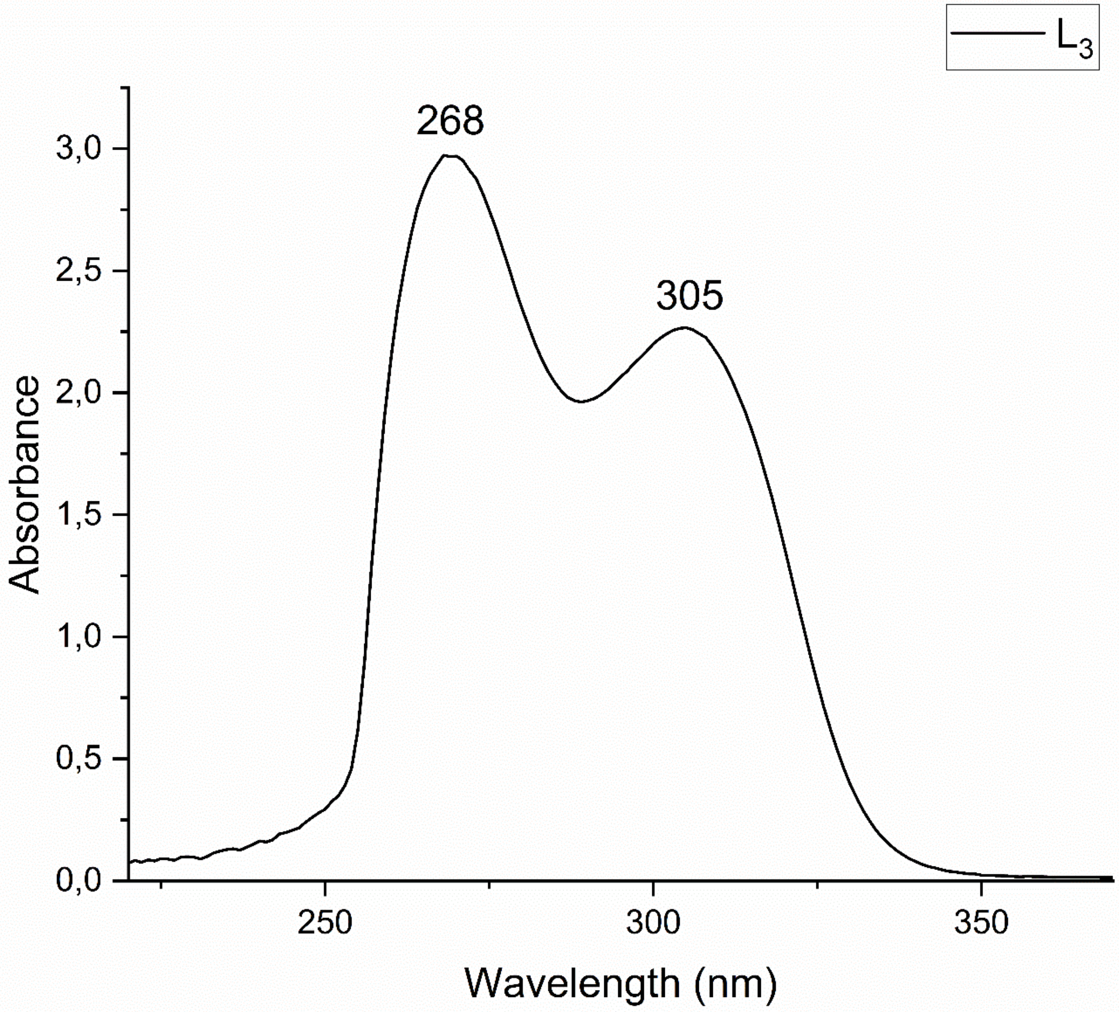

Figure S19. UV-VIS spectrum in DMSO of $\mathbf{L_3}$ at 2.5×10^{-4} M

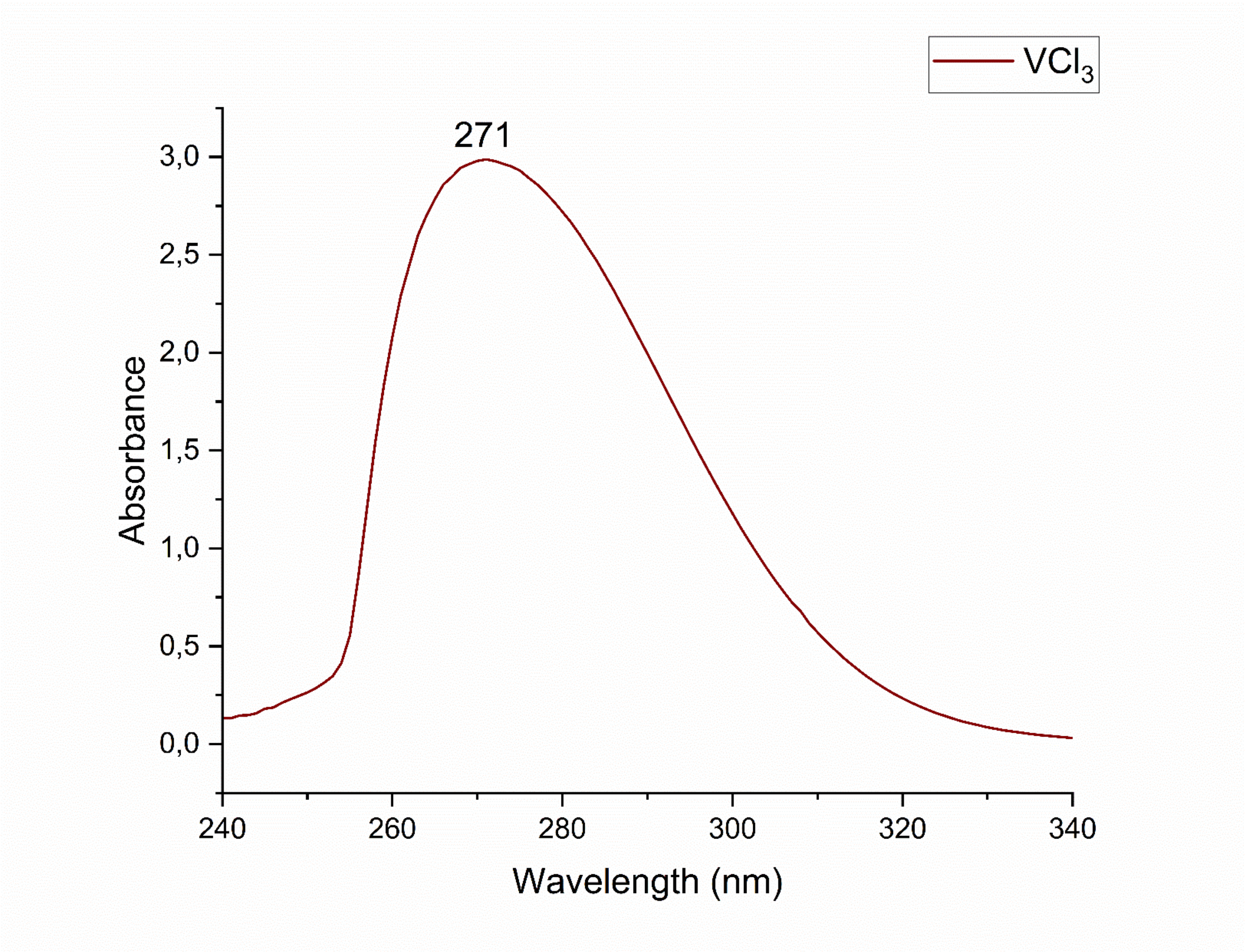

Figure S20. UV-VIS spectrum in DMSO of **VCl_3** in the range of 240 – 340 nm at $2.5x10^{-4}$ M

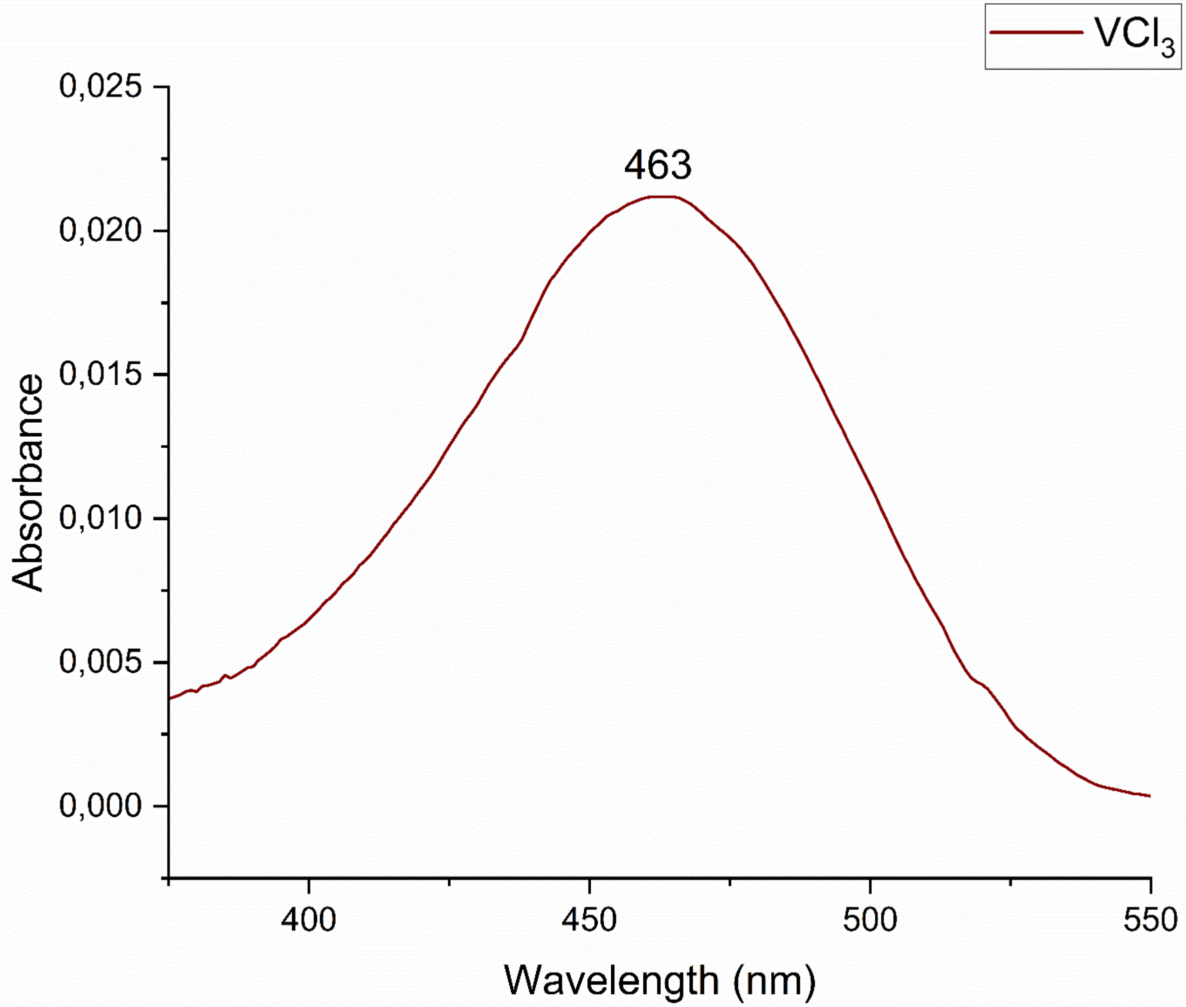

Figure S21. UV-VIS spectrum in DMSO of $\mathbf{VCl_3}$ in the range of 420 – 550 nm at $1x10^{-3}$ M

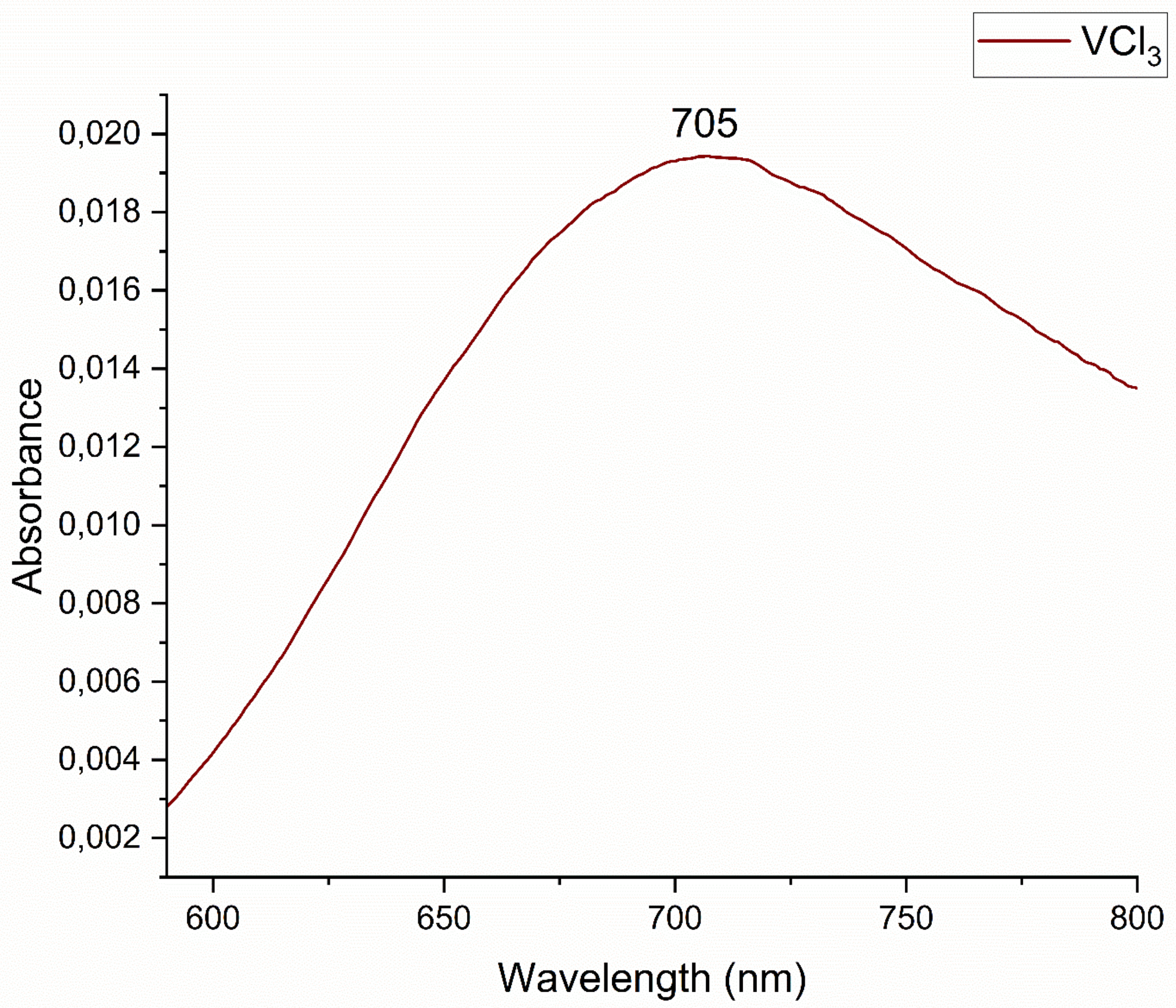

Figure S22. UV-VIS spectrum in DMSO of **VCl_3** in the range of 590 – 800 nm at $1x10^{-3}$ M

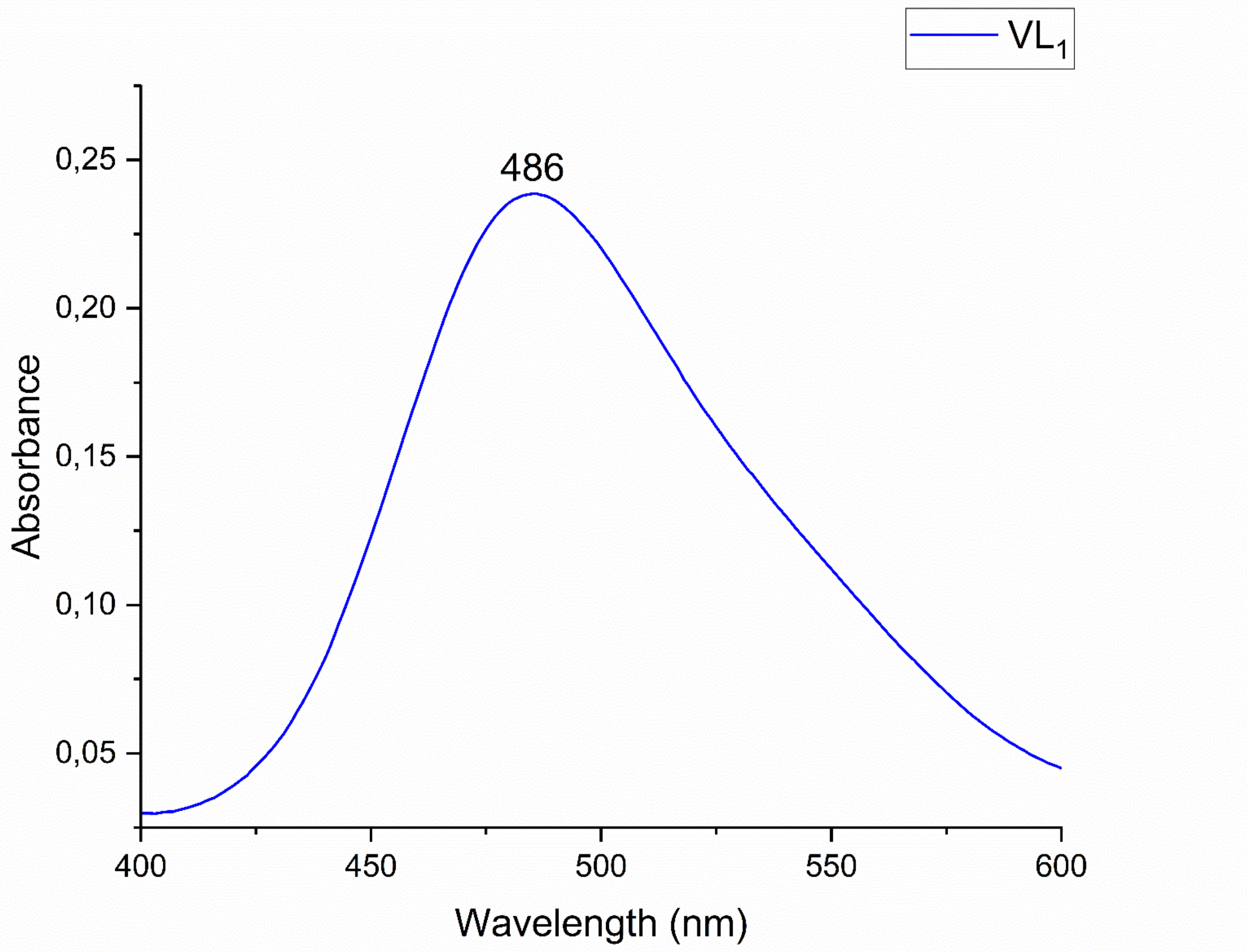

Figure S23. UV-VIS spectrum in DMSO of **VL_1** in the range of 400 – 600 nm at $1x10^{-3}$ M

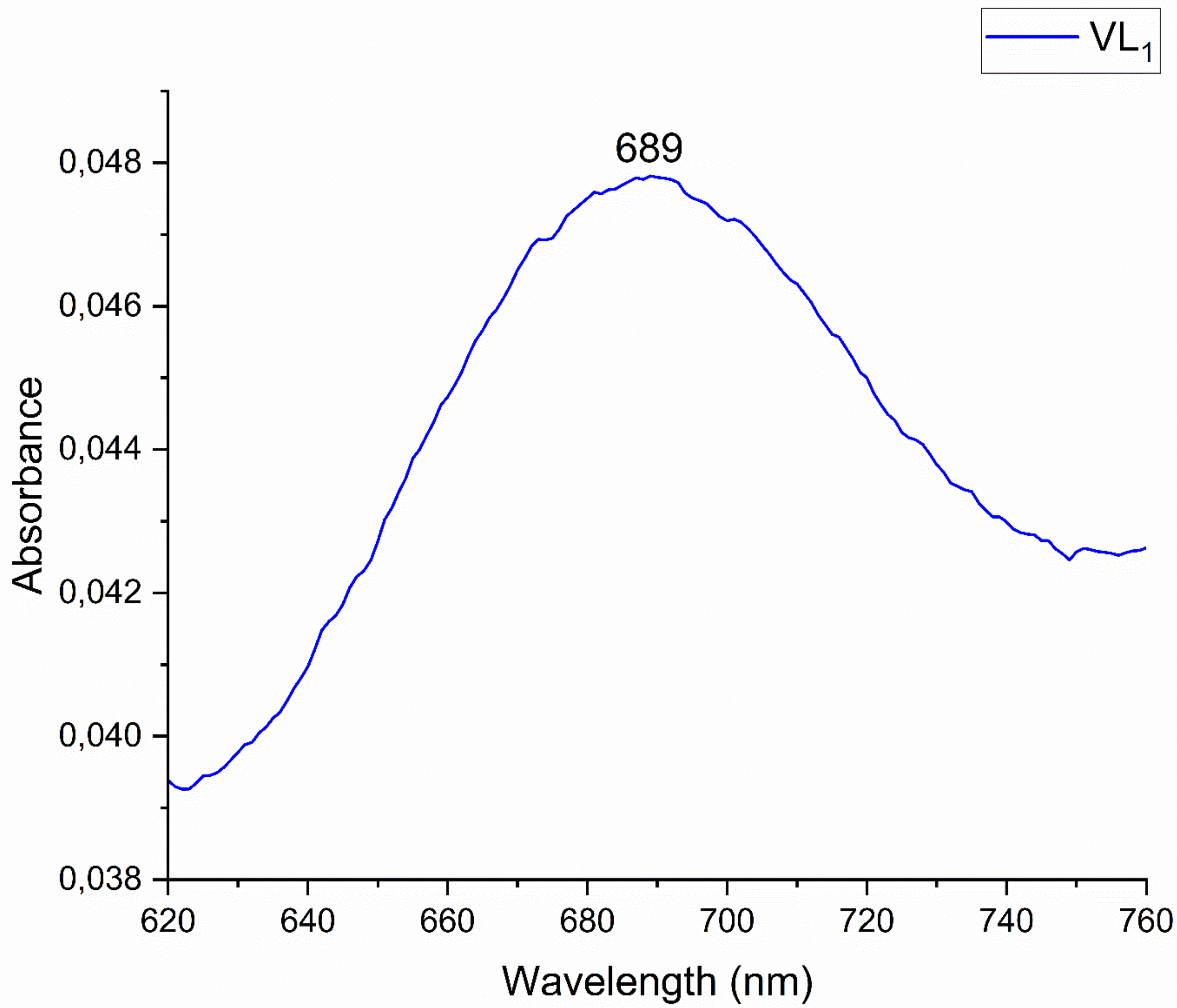

Figure S24. UV-VIS spectrum in DMSO of **VL_1** in the range of 620 – 760 nm at $1x10^{-3}$ M

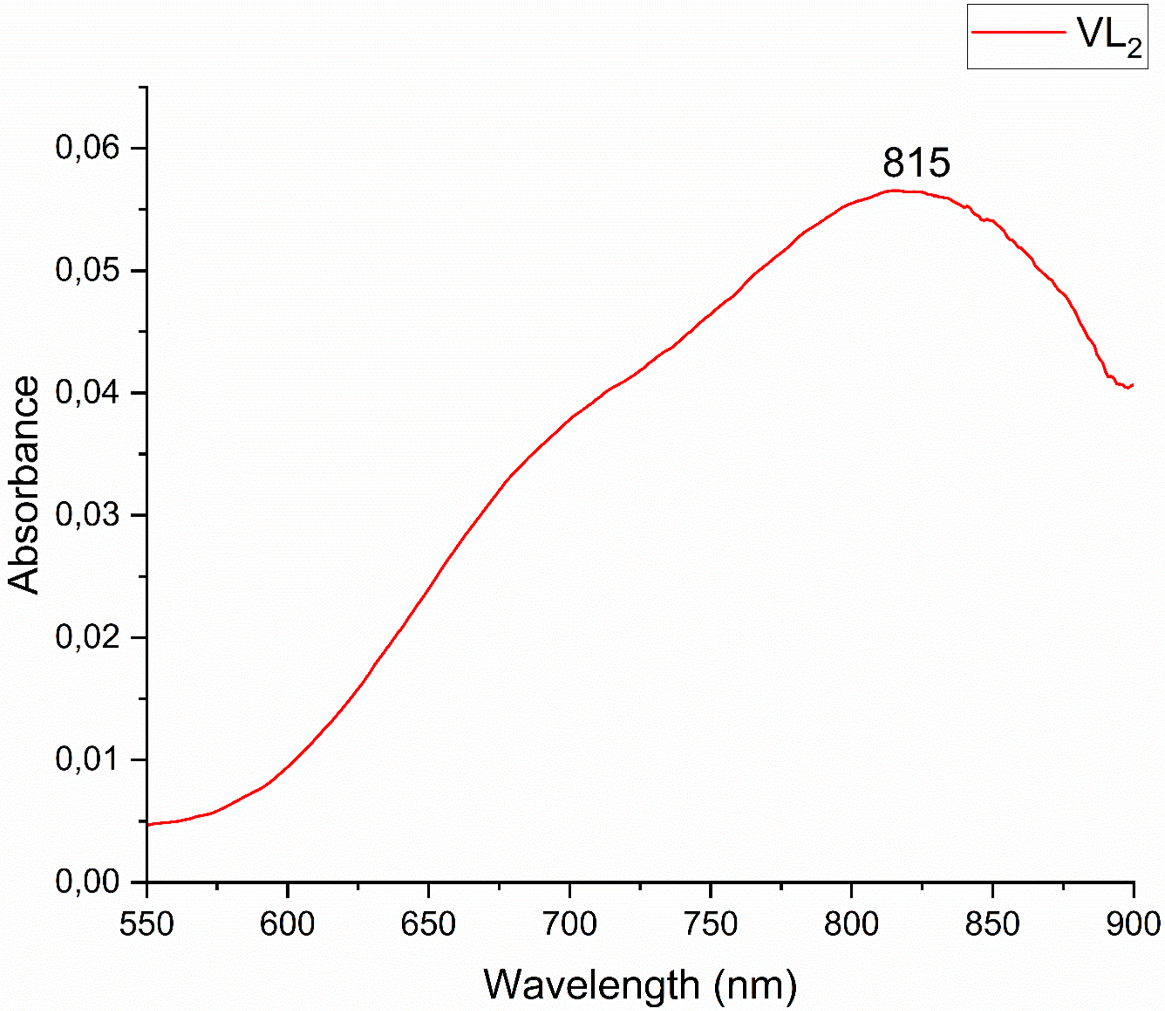

Figure S25. UV-VIS spectrum in DMSO of **VL_2** in the range of 550 – 900 nm at $1x10^{-3}$ M

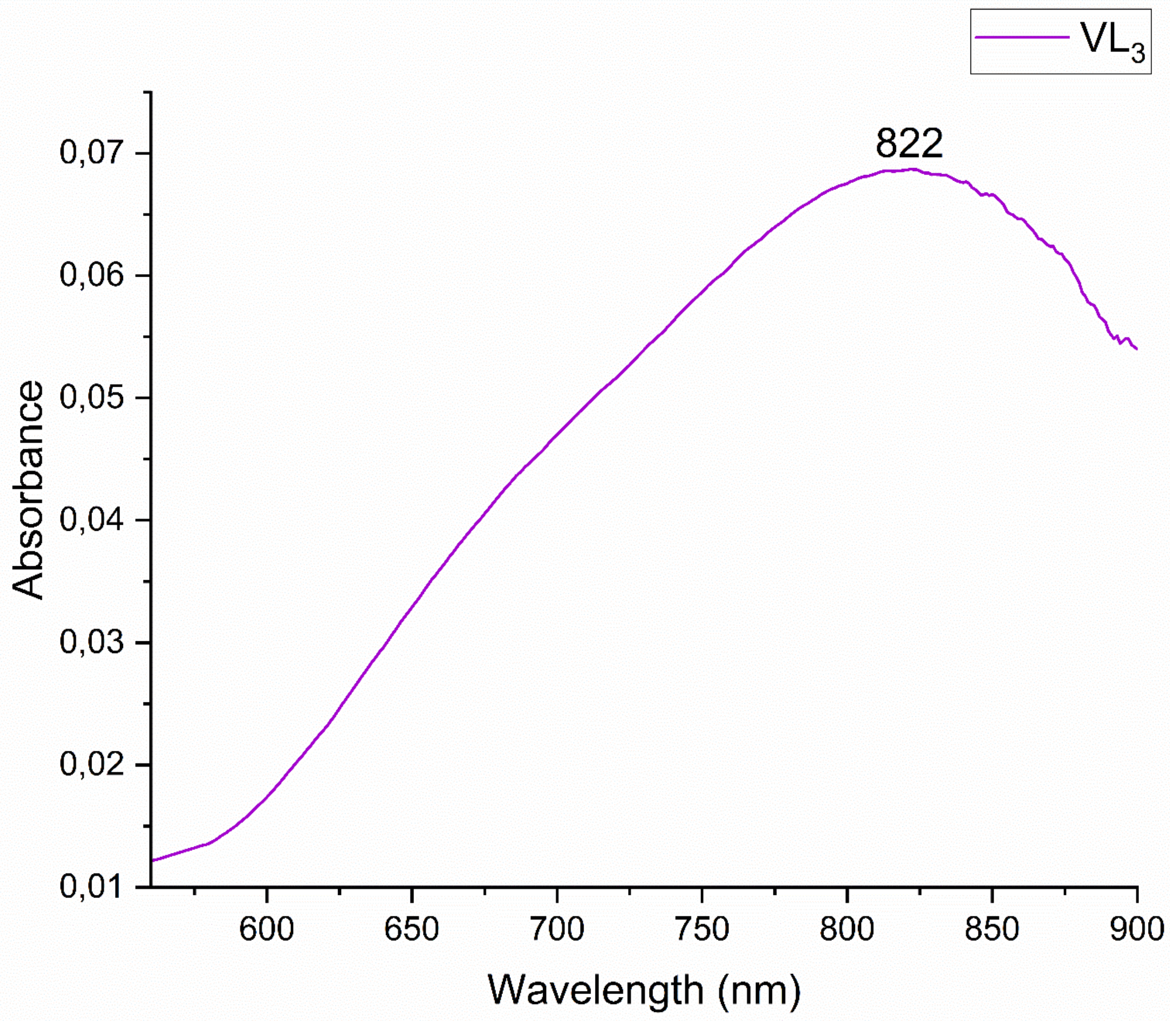

Figure S26. UV-VIS spectrum in DMSO of $\mathbf{VL_3}$ in the range of 580 – 900 nm at $1x10^{-3}$ M

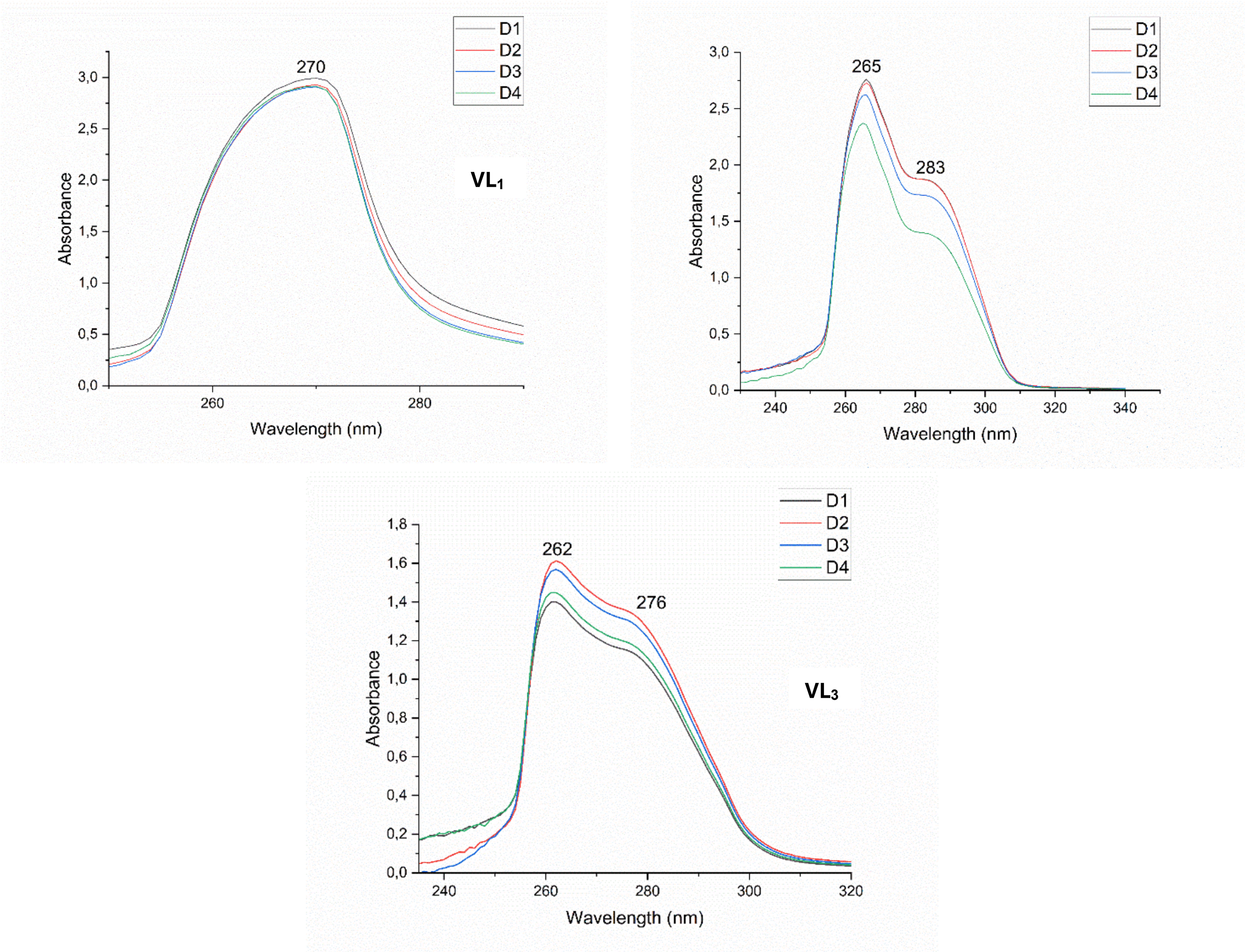

Figure S27. UV-VIS spectrum of the stability in DMSO of **VL$_1$-VL$_3$** at 2.5x10^{-4} M (D: day).

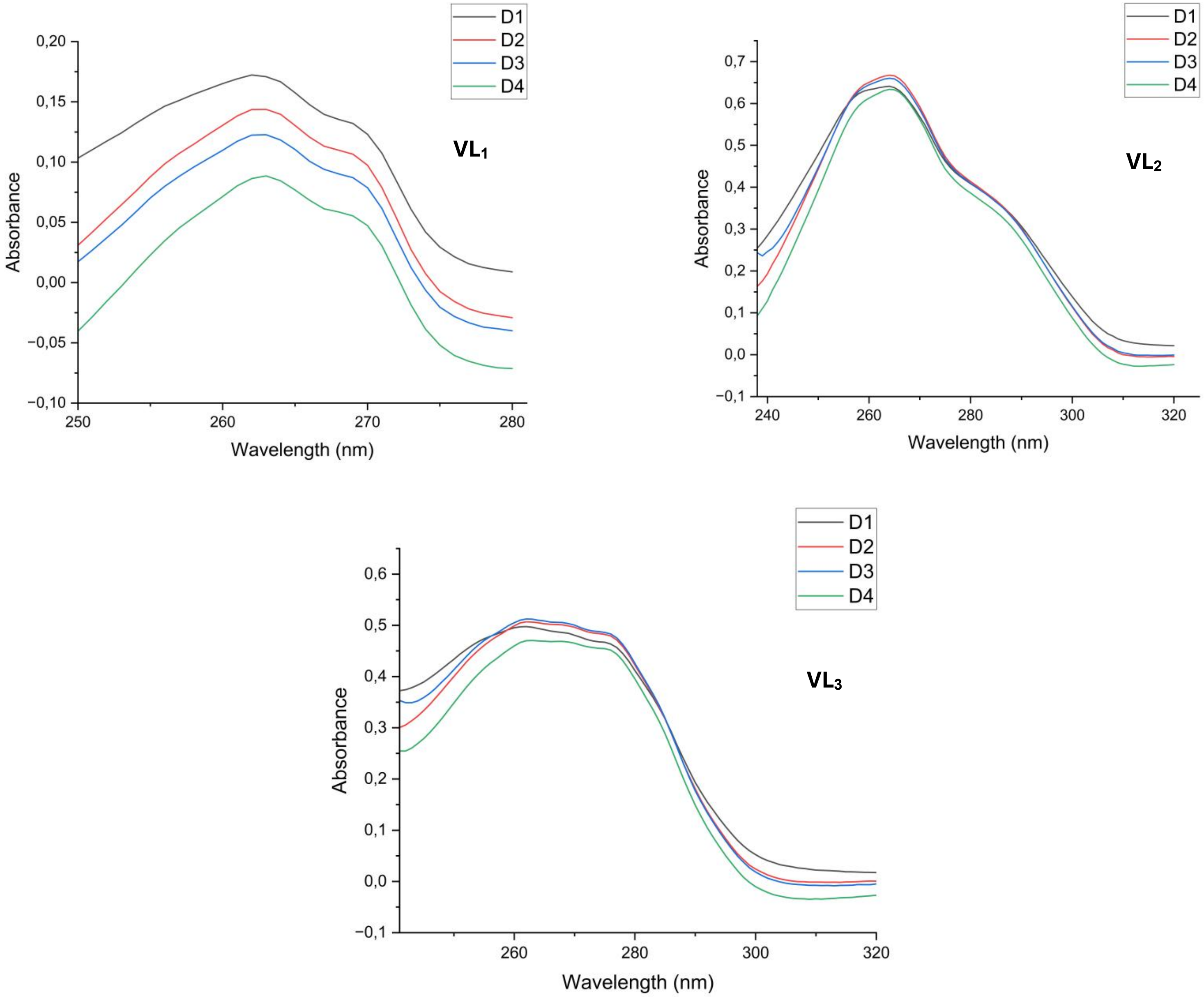

Figure S28. UV-VIS spectrum of the stability in H_2O:DMSO (95:5) of **VL$_1$-VL$_3$** (D: day).

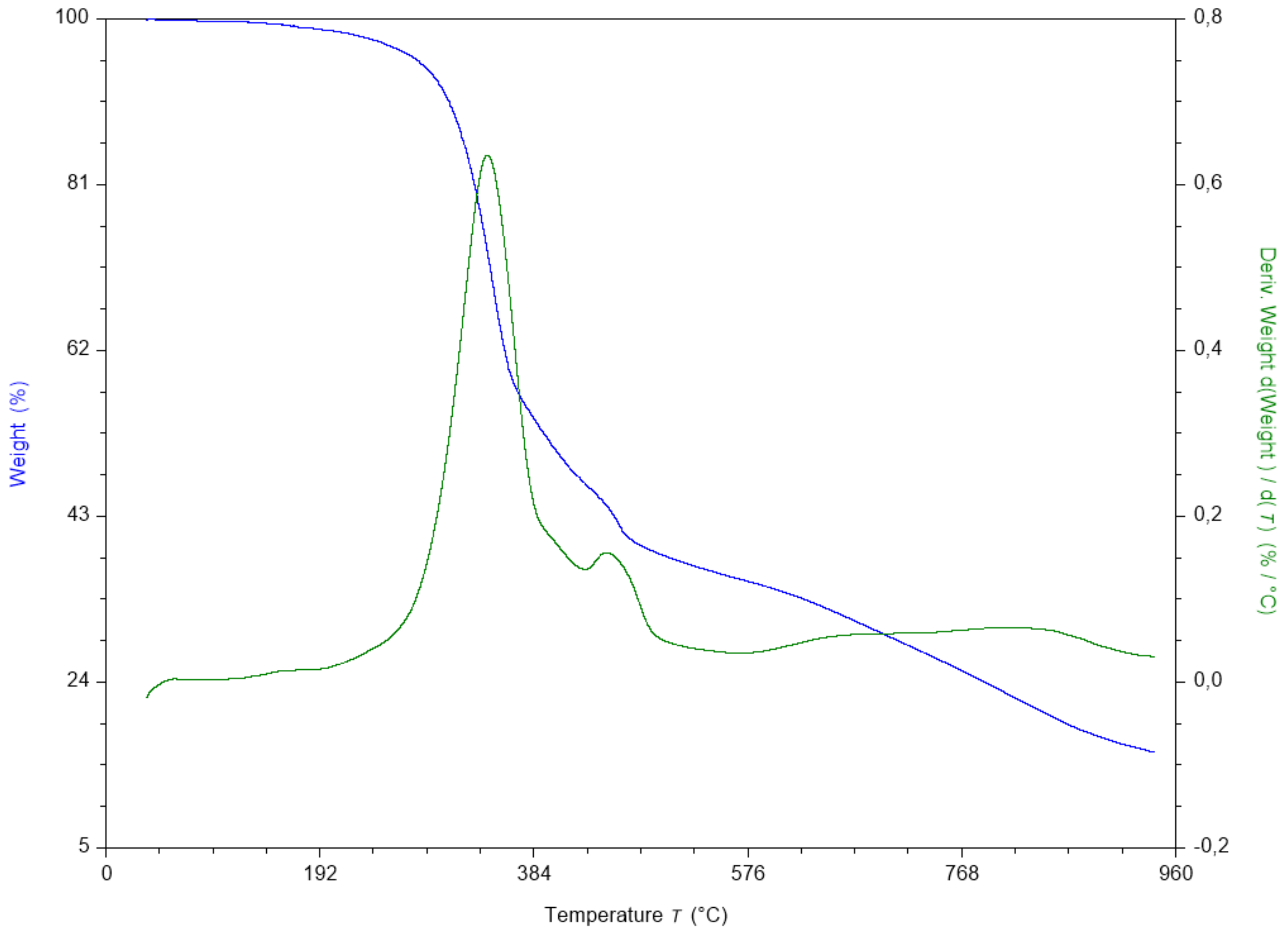

Figure S29. Thermogravimetric analysis (TGA) and derivative thermogravimetric (DTG) of **VL$_1$** in nitrogen atmosphere

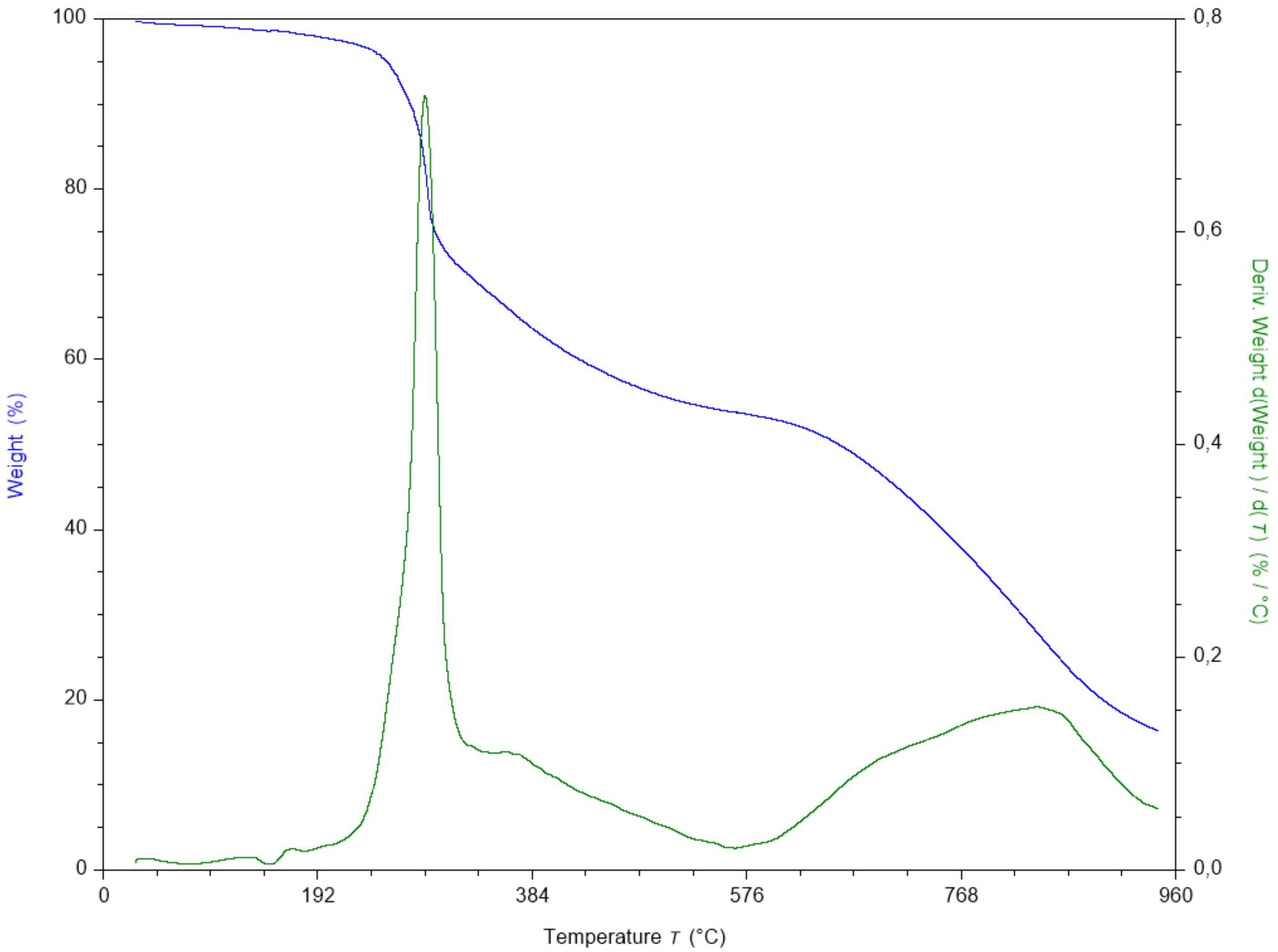

Figure S30. Thermogravimetric analysis (TGA) and derivative thermogravimetric (DTG) of **VL$_2$** in nitrogen atmosphere

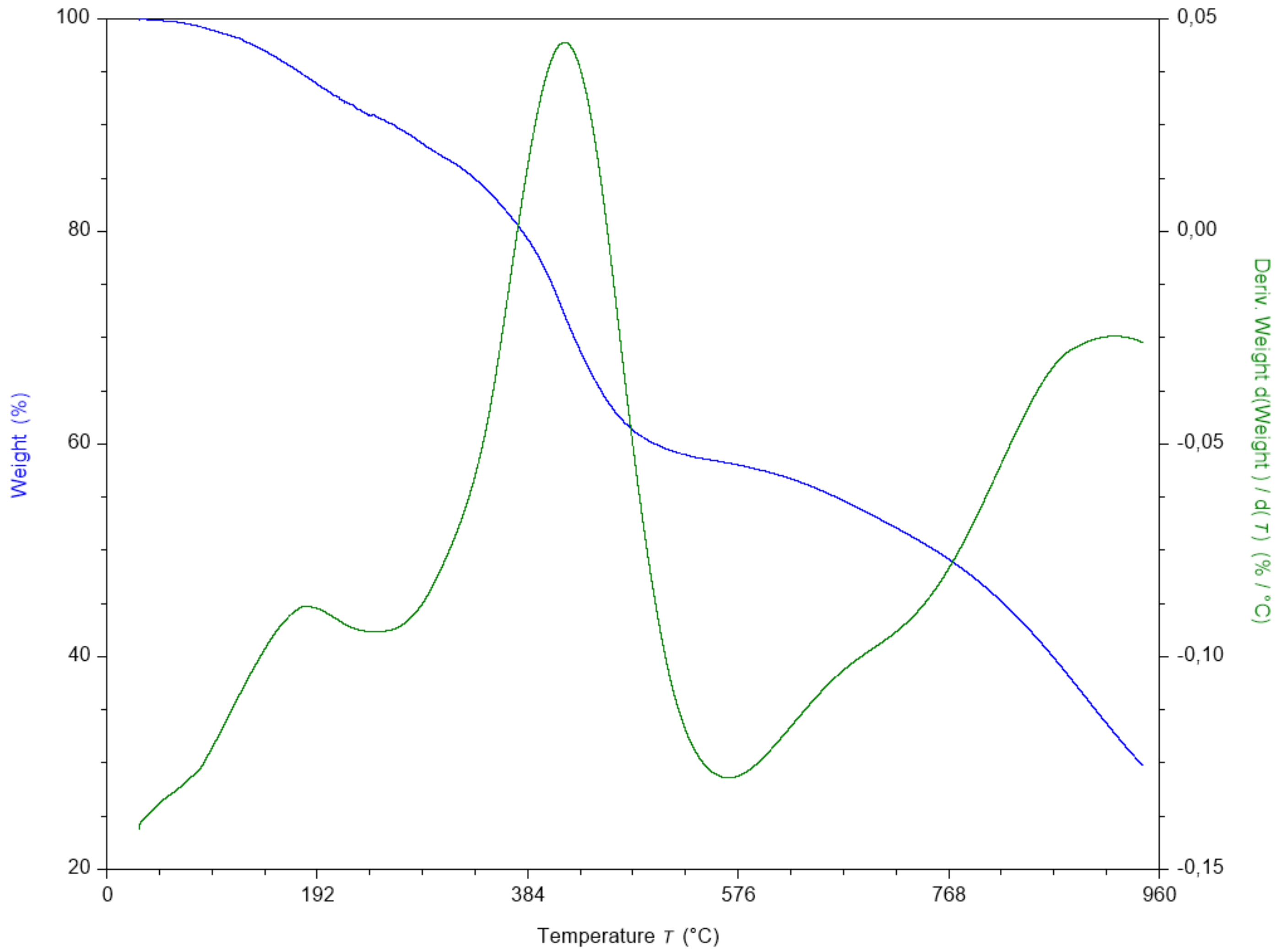

Figure S31. Thermogravimetric analysis (TGA) and derivative thermogravimetric (DTG) of **VL_3** in nitrogen atmosphere

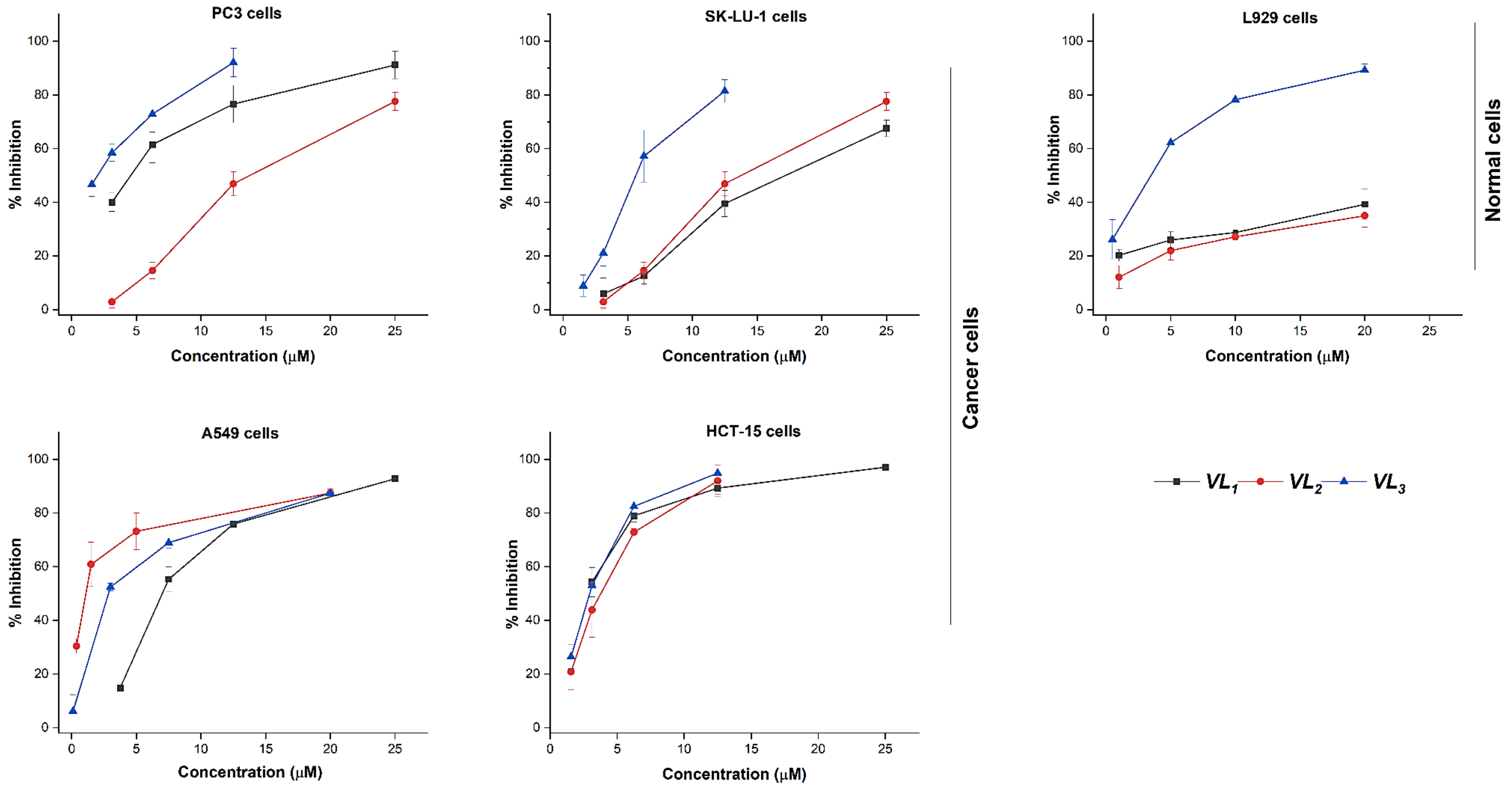

Figure S32. Dose-response curves for vanadium(III) complex ***VL_1 – VL_3***

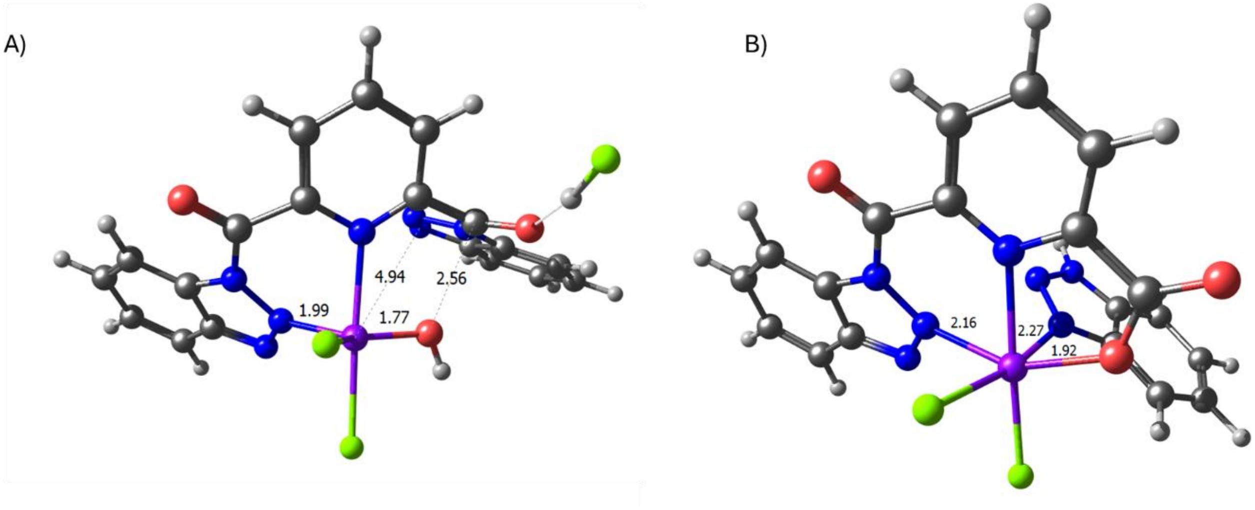

Figure S33. Cyclization process in the presence of water. A) Initial interaction involving dissociation of water and subsequent formation and release of HCl. B) Nucleophilic attack by the hydroxyl group resulting in cyclization and protonation of the triazole ligand. Distances in Armstrongs

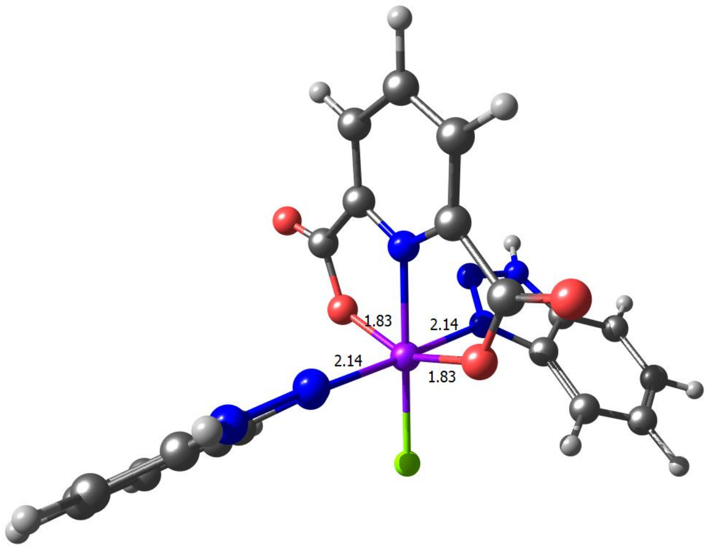

Figure S34. Second Cyclization process in the presence of water (distances in Armstrongs)

Table S1. Crystal data, data collection, and refinement information of **VL_3D**

**Crystal data**	
Chemical formula	$C_{19}H_{13}N_7O_5V$
M_r	470.30
Crystal system, space group	Orthorhombic, *Pbna*
Temperature (K)	298
a, *b*, *c* (Å)	12.9576 (13), 19.506 (2), 8.0011 (8)
V (Å^3)	2022.3 (4)
Z	4
Radiation type	Cu *K*α
μ (mm^{-1})	4.54
**Data collection**	
Diffractometer	SuperNova, Dual, Cu at zero, Atlas
Absorption correction	Multi-scan<br>*CrysAlis PRO* 1.171.41.119a (Rigaku Oxford Diffraction, 2021)
T_{min}, T_{max}	0.820, 1.000
No. of measured, independent and observed [$I > 2\sigma(I)$] reflections	7437, 2083, 1839
R_{int}	0.036
$(\sin \theta/\lambda)_{max}$ (Å^{-1})	0.630

Refinement	
$R[F^2 > 2\sigma(F^2)]$, $wR(F^2)$, S	0.058, 0.170, 1.08
No. of reflections	2083
No. of parameters	147
H-atom treatment	H-atom parameters constrained
$\Delta\rho_{max}$, $\Delta\rho_{min}$ (e Å^{-3})	0.40, −0.34

Table S2. Cytotoxic screening of ligands ***L_1* – *L_3*** and their vanadium(III) complexes ***VL_1* – *VL_3*** against cancer cell lines.

Compound	***Growth inhibition (%)***						
	**A549**	**SK-LU-1**	**HCT-15**	**PC3**	**K562**	**U251**	**MCF-7**
**L_1**	17.16	3.43	9.45	11.59	20.84	N.E.	N.E.
**VL_1**	79.43	83.87	92.07	91.72	34.21	48.29	6.46
**L_2**	N.E.	9.22	4.9	10.20	N.E.	N.E.	0.71
**VL_2**	98.27	86.49	94.62	100	41.35	50.55	35.13
**L_3**	N.E.	N.E.	2.8	4.43	N.E.	N.E.	N.E.
**VL_3**	92.76	89.01	100	100	32.56	58.44	N.E.

N.E. Not effect observed. The results shown are relative values normalized with negative controls.

Table S3. Log p values for ligands and complexes

Compound	Log P
**L_1**	0.3612
**VL_1**	0.0419
**L_2**	2.9206
**VL_2**	2.8732
**L_3**	2.2010
**VL_3**	2.1536
